# TGF-β Controls miR-181/ERK Regulatory Network during Retinal Axon Specification and Growth

**DOI:** 10.1371/journal.pone.0144129

**Published:** 2015-12-07

**Authors:** Sabrina Carrella, Sara Barbato, Ylenia D’Agostino, Francesco Giuseppe Salierno, Anna Manfredi, Sandro Banfi, Ivan Conte

**Affiliations:** 1 Telethon Institute of Genetics and Medicine, Via Campi Flegrei 34, Pozzuoli (Naples), 80078, Italy; 2 Medical Genetics, Dipartimento di Biochimica, Biofisica e Patologia Generale, Second University of Naples, via Luigi De Crecchio 7, 80138, Naples, Italy; University of Michigan, UNITED STATES

## Abstract

Retinal axon specification and growth are critically sensitive to the dosage of numerous signaling molecules and transcription factors. Subtle variations in the expression levels of key molecules may result in a variety of axonal growth anomalies. miR-181a and miR-181b are two eye-enriched microRNAs whose inactivation in medaka fish leads to alterations of the proper establishment of connectivity and function in the visual system. miR-181a/b are fundamental regulators of MAPK signaling and their role in retinal axon growth and specification is just beginning to be elucidated. Here we demonstrate that miR-181a/b are key nodes in the interplay between TGF-β and MAPK/ERK within the functional pathways that control retinal axon specification and growth. Using a variety of *in vivo* and *in vitro* approaches in medaka fish, we demonstrate that TGF-β signaling controls the miR-181/ERK regulatory network, which in turn strengthens the TGF-β-mediated regulation of RhoA degradation. Significantly, these data uncover the role of TGF-β signaling *in vivo*, for the first time, in defining the correct wiring and assembly of functional retina neural circuits and further highlight miR-181a/b as key factors in axon specification and growth.

## Introduction

Eye formation in vertebrates requires a series of morphogenetic events orchestrated by the interplay between a number of signaling pathways and transcription factors that regulate specific genetic programs. The concerted action of numerous cell-intrinsic and -extrinsic factors is required for retinal progenitor cell growth, elaboration of distinct neural cell types, spatial patterning, and axonal connectivity [1]. Among a number of evolutionarily conserved signaling pathways, the transforming growth factor-β (TGF-β) superfamily of secreted ligands are known to mediate important functions in the proliferation and cell death during retinal development [2–4]. However, there are indications of additional functions of TGF-β signaling in retinal patterning that remain to be elucidated.

TGF-β signaling acts through two main mechanisms, i.e. the Smad-dependent and the Smad-independent cascade. The Smad-dependent cascade is mediated through the direct regulation of Smad proteins by TGF-β receptors [5, 6]. Smad activation results in their accumulation in the nucleus and the transcriptional regulation of target genes. The Smad-independent cascade relies on the ability of TGF-β receptors to activate other signaling pathways, such as several kinase signaling pathways [7], that help to define the responses to TGF-β factors. Indeed, the combinatorial usage of Smad-dependent mechanism components and Smad-independent signaling mechanisms are fundamental to reinforce, attenuate, or modulate downstream cellular responses giving rise to the wide spectrum of processes modulated by TGF-β signaling [7, 8]. For instance, in neurons, TGF-β signaling contributes to axon specification and growth through distinct Smad-independent mechanisms [9, 10] and its absence induces both axonal and dendritic degeneration [11].

Recently, TGF-β signaling has been shown to play a regulatory role in microRNA (miRNA) regulation through a Smad-dependent mechanism [12, 13]. miRNAs are a class of 20- to 25-nucleotide small noncoding RNA molecules that post-transcriptionally regulate gene expression. We recently demonstrated that the miRNAs miR-181a/b act as key regulators of retinal axon specification and growth through negative modulation of MAPK/ERK signaling [14]. Previous *in vitro* studies reported that miR-181a/b family members could be regulated by TGF-β at both transcriptional and processing level, depending on cell type [15–17]. However, there is no information on the TGF-β-mediated regulation of miR-181a/b *in vivo* and whether this can have an impact in vertebrate retinal axon specification and growth. Interestingly, the TGF-β/BMP and MAPK/ERK pathways have opposite effects on axon/dendrite specification and growth [18] through mechanisms that are yet unknown.

The present study aims at elucidating whether TGF-β may indeed control the MAPK/ERK pathway in the process of retinal axon specification and growth *in vivo* and whether this action could be mediated through the modulation of miR-181a/b expression. To reach this goal, we relied on the medaka fish [*Oryzias latipes* (Ol)] model organism whose genome harbors four different copies of the miR-181a/b clusters, namely on chromosome 4, chromosome 9, chromosome 17 and on a locus that is yet unassigned (Ultracontig105) [14]. We showed that TGF-β signaling exerts an inhibitory action on the MAPK/ERK pathway through direct activation of miR-181a/b. In addition, we identified a novel regulatory network composed by TGF-β, miR-181a/b and the MAPK pathway that works in concert with the TGF-β-mediated regulation of RhoA degradation in retinal axon specification. This study is the first to reveal *in vivo* that miR-181a/b are part of the TGF-β genetic network, adding knowledge to the repertoire of TGF-β activities during the course of axon specification and growth in vertebrate eye development.

## Results

### TGF-β signaling regulates the levels of mature miR-181a/b in the retina

**Fig 1:**
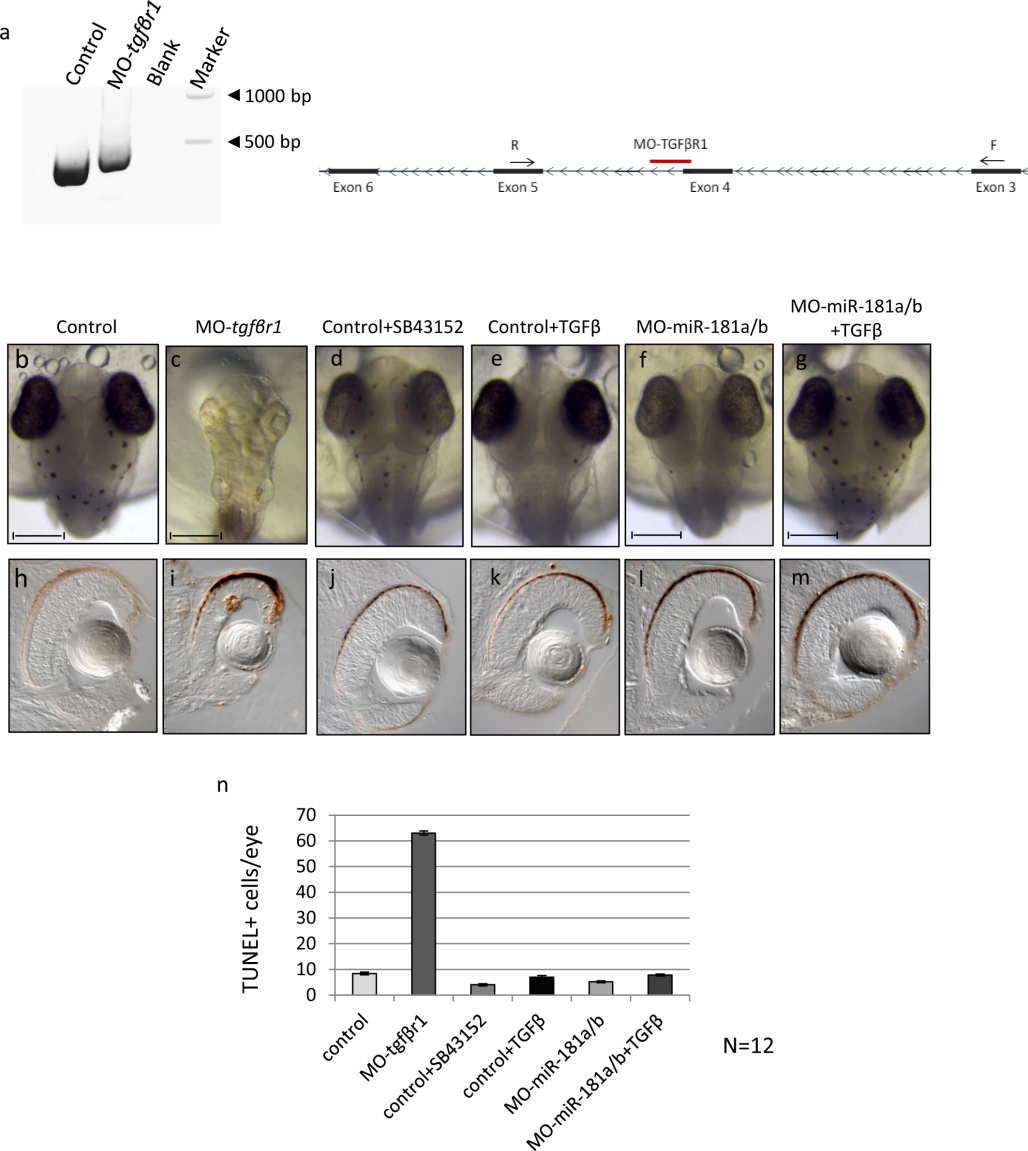
TGF-β pathway down-regulation from early phases of medaka fish embryo development determines alteration of programmed cell death programs in the retina. **(a)** MO-*tgfβr1* is designed to sterically block the fourth intron-exon splice donor site of the *tgfβr1* transcript, causing a partial retention of the intronic sequence as shown by PCR analysis. **(b-g)** Control (b), MO-*tgfβr1* (c), SB43152 (d) and TGF-β- (e) treated control, MO-miR-181a/b (f) and TGF-β treated MO-miR-181a/b (g) medaka fish embryos at stage 32. The MO-*tgfβr1* injected embryos showed a phenotype characterized by abnormal body and head structures, including microphthalmia. **(h-m)** Alteration of the TGF-β pathway from the early stages of development caused an increase of retina cell death as shown by TUNEL assay (h, i). Administration of drugs that lead to a TGF-β pathway down-regulation (j, SB43152) or increase (k, TGF-β) from stage 30 onwards did not cause cell death alteration in control medaka retina. Similarly, no significant alterations were found in MO-miR-181a/b- (l) and TGF-β-treated MO-miR-181a/b (m) retinas. **(n)** Quantification of TUNEL positive cells; N = 12 eyes were analyzed for each treatment.

In a previous study, we demonstrated that miR-181a/b are required for retinal axon specification and growth through the modulation of MAPK/ERK signaling [14]. We now hypothesize that miR-181a/b may act as nodes in a signaling network involving TGF-β and MAPK/ERK pathways. To validate the above hypothesis and to identify yet unexplored roles of TGF-β in eye development and function, we carried out a detailed analysis on the role of TGF-β-mediated control of miR-181a/b expression in retinal axon specification and growth. We figured that TGF-β could regulate miR-181a/b levels either by SMAD-mediated transcriptional regulation (i.e. by increasing the transcription of the corresponding primary (pri-) miRNA), or by the regulation of miRNA maturation through SMAD2/3-binding to the Drosha/DGCR8 complex [13]. If either or both of the above scenarios are true, then inhibition of the TGF-β pathway activity should decrease miR-181a/b expression in medaka fish eyes, where miR-181a/b are highly expressed [14]. To investigate this possibility, we interfered with TGF-β Receptor 1 (Tgfβr1) protein synthesis using a morpholino (MO)-based knockdown approach. To this end, a specific MO oligonucleotide (MO-*tgfβr1*, see S1 Table) was designed to sterically block the fourth splice donor site of the *Tgfβr1* transcript (Fig 1A, S1 Supporting Text).

The MO-*tgfβr1* was injected into fertilized one-cell medaka fish embryos. MO-mediated down-regulation of *tgfβr1* induced a phenotype characterized by abnormal body and head structures, including microphthalmia (Fig 1B and 1C). This phenotype was in accordance with what was previously shown in other TGF-β pathway component ablation in various model systems, reinforcing the importance of this signaling pathway for ocular development, starting from early eye morphogenesis, retinal pigment epithelium fate determination, retina programmed cell death (PCD) and neurogenesis [2, 4, 19, 20]. Indeed, TUNEL assay revealed a strong increase of cell death in the eyes of MO-*tgfβr1* injected embryos, with respect to controls (Fig 1H and 1I). The severity of the phenotype caused by early knockdown of Tgfβr1 prevented us to properly investigate the possible role of TGF-β in miR-181a/b regulation.

**Fig 2:**
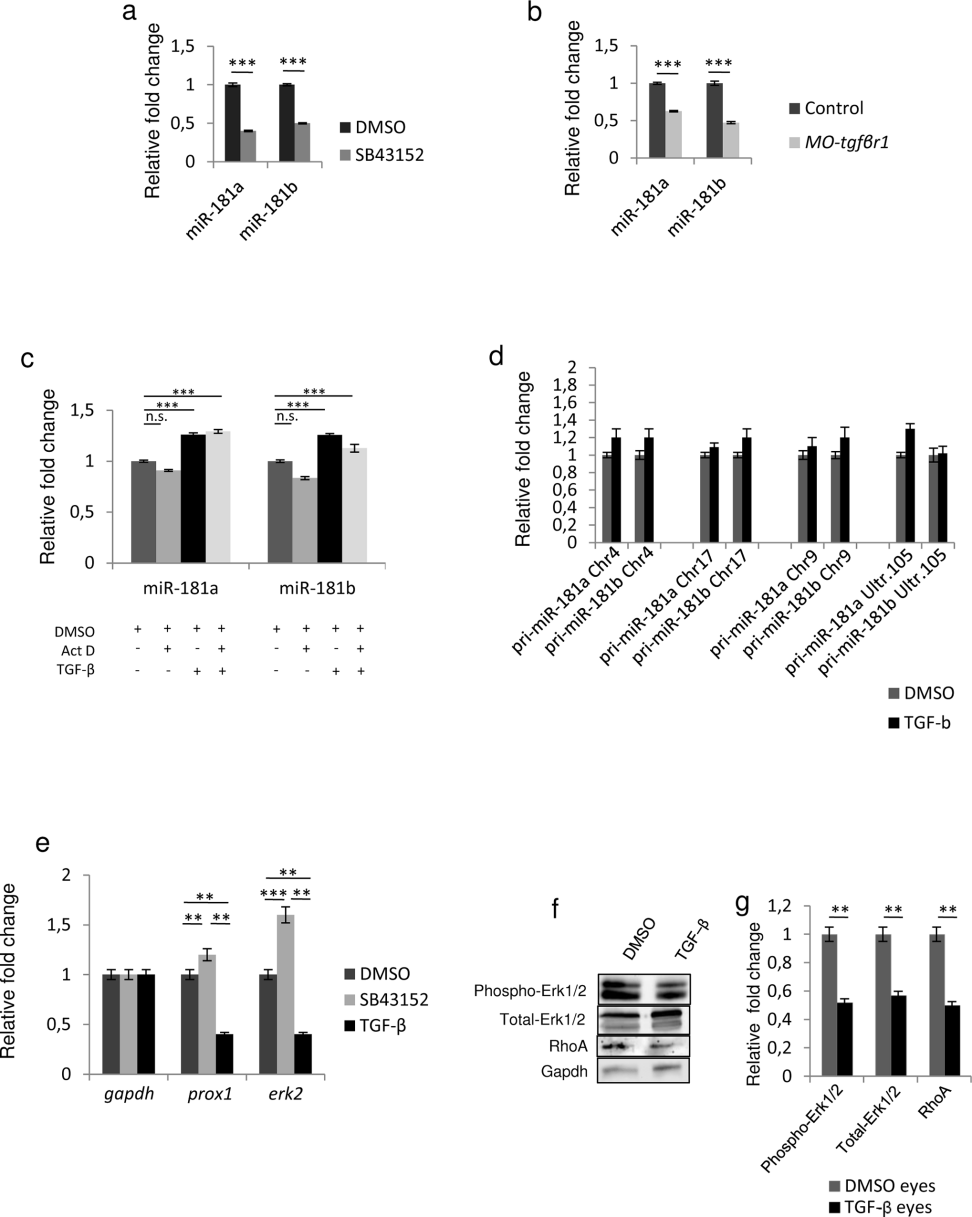
TGF-β signaling regulates mature miR-181a/b expression levels. **(a-b)** TGF-β pathway inhibition leads to a decrease in the expression levels of mature miR-181a and miR-181b. **(a)** Administration of SB432542, a TGF-β receptor inhibitor, induced a decrease of miR-181a and miR-181b mature forms in St32 eyes with respect to DMSO treatment, as detected by Taqman assays. The miR-181a and miR-181b reduction was comparable with that observed in the morpholino-mediated inhibition of the TGF-β pathway (MO-*tgfβr1*) at St32 **(b)**. Data are means +SEM. ***, P <0.001 (t-tests). **(c-d)** TGF-β treatment (10ng/ml) leads to increased levels of miR-181a and miR-181b mature forms in medaka fish St32 eyes in a transcription-independent manner. **(c)** Administration of TGF-β (10ng/ml) for 24 h (from St30 to St32) led to the increase of mature miR-181a and miR-181b in St32 eyes, as assessed by Taqman assays. Co-treatment with TFG-β and actinomycin D for 24 h (from St30 to St32) did not alter the TGF-β effect on mature miR-181a/b levels. These results indicate that the TGF-β effect on miR-181a/b expression is not transcription-dependent. Data are means +SEM. ***, P <0.001 (two-way ANOVA). **(d)** qRT-PCR on RNA extracted from DMSO- and TGF-β-treated St32 medaka fish eyes for all the pri-miR-181a and pri-miR-181b transcripts derived from the different genomic loci present in the medaka fish genome. After 24 h (from St30 to St32) of TGF-β treatment (10ng/ml), there were no significant changes in pri-miR-181a/b levels with respect to DMSO treatment. **(e)** qRT-PCR on RNA extracted from DMSO-, SB432542- and TGF-β-treated St32 medaka fish eyes. In the SB432542-treated eyes the decrease of miR-181a/b levels led to increased *prox1* and *erk2* transcript levels, whereas in TGF-β-treated eyes the miR-181a/b increase was accompanied by reduced transcript levels of both *prox1* and *erk2*. Data are means +SEM. **P <0.01; ***, P <0.001 (Two-way ANOVA). **(f-g)** Representative Western blotting (f) and corresponding quantification (g), showing a decrease of total-, phospho-Erk2 proteins and of its downstream target RhoA in TGF-β-treated St32 medaka fish eyes. Data are means +SEM. **P <0.01 (t-tests).

Therefore, we decided to use a different strategy that allowed us to inhibit the TGF-β pathway from the onset of miR-181a/b expression [Stage (St) 30 that corresponds to the beginning of retinal ganglion cells (RGCs) differentiation] onwards [14]. SB43152 is a specific inhibitor that targets TGF-β receptors and thereby inhibits SMAD2 and SMAD3 phosphorylation [21]. SB43152 treatment from St30 onwards allowed us to avoid the inhibition of the earlier functions of the TGF-β pathway in retinal development. Indeed, SB43152 treatment gave rise to a milder phenotype with respect to MO-*tgfβr1*-injected embryos (Fig 1D) and did not alter retinal cell death, as assessed by TUNEL staining (Fig 1J–1N). After 24h of SB43152 treatment (St32, onset of amacrine cells differentiation), hydrolysis probes (Taqman) assay revealed a significant reduction in mature miR-181a and miR-181b levels in total eye RNA (Fig 2A), comparable with that observed in the morpholino-mediated inhibition of the TGF-β pathway (Fig 2B). Conversely, we found that TGF-β treatment, from St30 to St32, led to increased levels of the mature forms of both miR-181a and miR-181b in the eye, as compared to DMSO-treated controls (Fig 2C). As previously mentioned, these higher expression levels might be due to either increased transcription or enhanced post-transcriptional miRNA maturation exerted by TGF-β. However, a 24-h (from St30 to St32) co-treatment with TGF-β and actinomycin D, a general inhibitor of transcription, did not prevent the increase in mature miR-181a and miR-181b expression (Fig 2C), thus suggesting that the TGF-β–mediated up-regulation of miR-181a/b is exerted at post-transcriptional level. Moreover, we observed no significant modifications in pri-miR-181a and pri-miR-181b expression in TGF-β–treated wild-type eyes (Fig 2D). Overall, these data support the hypothesis that TGF-β activates miR-181a/b expression *in vivo* mainly through SMAD2/3-mediated enhanced processing of miRNAs.

We recently demonstrated that miR-181a/b are able to negatively modulate MAPK/ERK signaling, which in turn leads to RhoA reduction and ensures proper neuritogenesis in both amacrine cells and retinal ganglion cells (RGCs) via local cytoskeletal rearrangement [14]. Intense cytoskeletal rearrangement allows growth cone structures to protrude, enabling the specification and formation of a single tau-1 positive axon. It has been demonstrated that axon formation takes place from the growth cone with a more dynamic and labile actin network [22]. RhoA is a GTPase protein involved in actin retraction. Increased levels of RhoA have been described to lead to axon growth cone collapse or turning [22–25]. Conversely, local degradation of RhoA alters actin dynamics, thus enhancing axon specification and outgrowth [22]. Recently, TGF-β has been reported to have a role in RhoA degradation in brain neurons during axon specification [10]. As TGF-β/BMP and MAPK/ERK pathways have opposite effects on axon/dendrite specification and growth [18, 26–28], we hypothesized that miR-181a/b could represent a link between TGF-β and MAPK/ERK signaling in retinal development. Indeed, miR-181a/b down-regulation upon SB43152 treatment is accompanied by an increase in transcript levels of *prox1* and *erk2* (Fig 2E), two validated miR-181a/b targets [29, 30]. Consistently, the positive action of TGF-β on the post-transcriptional maturation of miR-181a/b translated into an opposite effect (i.e., a decrease) on the expression of these miR-181a/b targets (Fig 2E). The decrease of *erk2* transcript levels induced by TGF-β administration was paralleled by a reduction of total- and phospho-Erk2 protein levels, as well as of its downstream target, RhoA (Fig 2F and 2G).

### TGF-β signaling regulates retinal axon specification and growth by modulating RhoA levels

**Fig 3:**
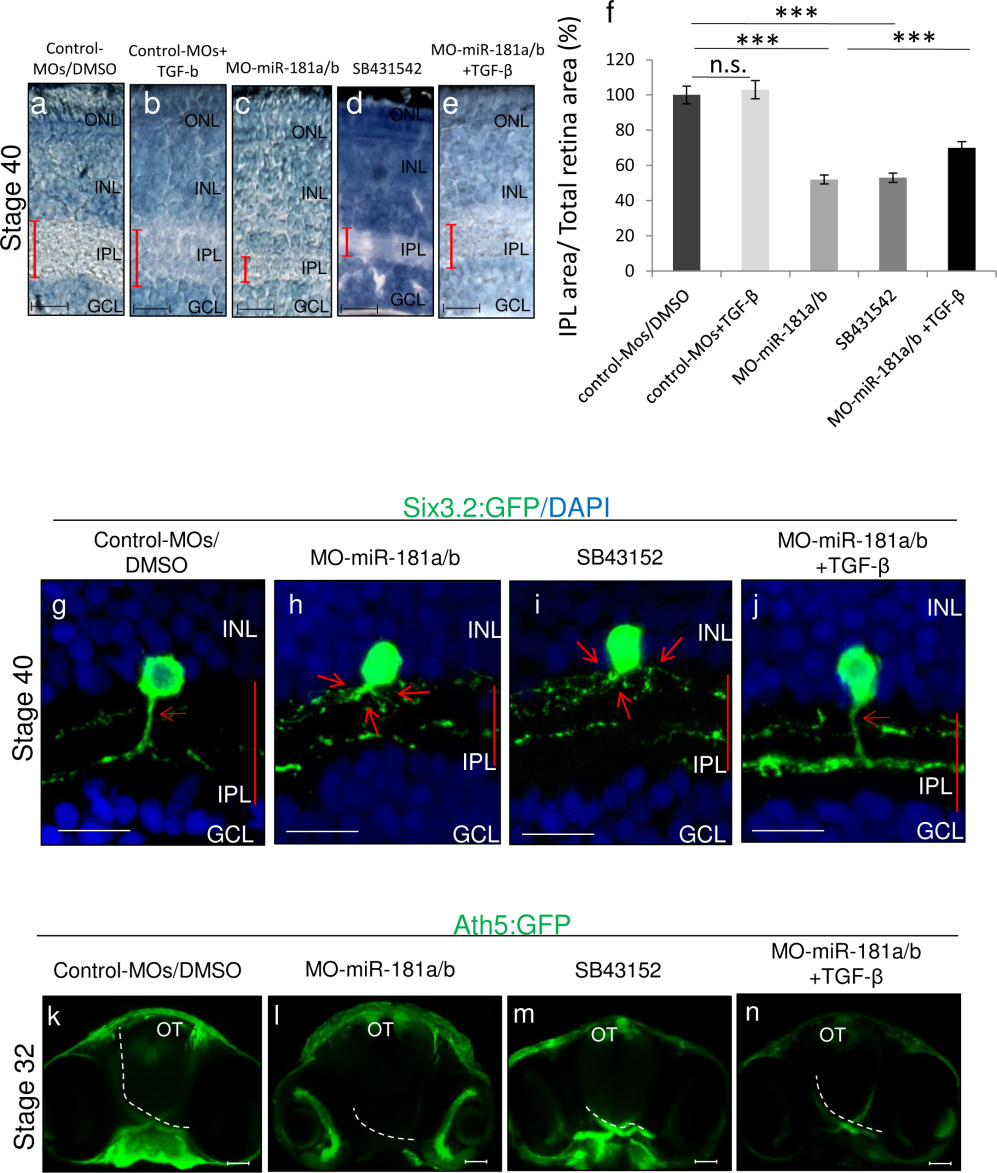
TGF-β signaling modulates miR-181a/b action in the assembly of retinal circuitry. **(a-e)** Retinal frontal sections of St40 DMSO-treated (a), TGF-β-treated (b) control-MO medaka fish embryos, miR-181a/b morphant embryos (c), SB432542-treated embryos (d) and TGF-β-treated (e) miR-181a/b morphant medaka fish embryos processed for Richardson-Romeis staining. Red bars, IPL thickness. Scale bars: 20μm. **(f)** Quantitative analysis of IPL thickness indicated as the ratio in the central retina between the IPL area and total retinal area. Data are means ± SEM. ***, P <0.001 (one-way ANOVA). **(g-j)** Representative images of amacrine cells from St40 retinal sections of control-MOs (g), miR-181a/b morphant (h), SB432542-treated control (i) and miR-181a/b morphant treated with TGF-β (j) Six3:eGFP transgenic medaka fish embryos. Cell nuclei are stained with DAPI (blue). GFP (green signal) stains amacrine cell soma and neurites; red arrows, Six3 axon-like structure of amacrine cells; red bars, thickness of the IPL. SB432542 treatment (i) phenocopied the amacrine cell neuritogenesis defects observed in miR-181a/b morphants (h). Addition of TGF-β (from St30 to St40) to miR-181a/b morphants (j) was sufficient to rescue neuritogenesis defects of miR-181a/b morphant transgenic embryos. INL, inner nuclear layer; GCL, ganglion cell layer. Scale bars: 20μm. **(k-n)** Representative 2-D reconstruction of confocal images of St32 control-MOs (k), miR-181a/b morphant (l), SB432542-treated (m) and TGF-β-treated miR-181a/b morphant (n) Ath5:eGFP transgenic whole-heads. Dotted white lines mark optic nerve routes. Treatment of control-MOs embryos with 80μM SB432542 (m) phenocopied the miR-181a/b-morphant optic nerve length decrease (l). Addition of TGF-β for 24 h (from St30 to St32) to miR-181a/b morphants (n) was sufficient to rescue correct optic nerve growth in Ath5:eGFP morphant embryos. Scale bars: 50μm. OT, optic tectum.

The depletion of miR-181a/b activity during retinal axon specification and growth results in a phenotype characterized by a decrease in the inner plexiform layer (IPL) thickness, failure in amacrine cell axon-like specification and decrease in RGC axon length, due to increased activity of the MAPK/ERK pathway [14]. Consistently, since TGF-β signaling inhibition causes decrease in miR-181a/b mature levels with consequent increase in *erk2* levels, we found that SB43512 administration led to a retinal phenotype that recapitulated the miR-181a/b morpholino-mediated loss-of-function (Fig 3A, 3C, 3D, 3F, 3G–3I and 3K–3M). At St40, i.e. when the retina has completed its maturation, the IPL of SB43512-treated embryos was abnormally thin, similar to what was observed in miR-181a/b morphants (Fig 3C, 3D and 3F). As previously described [14], the reduced IPL thickness in miR-181a/b morphants was accompanied by the failure of specification of axon-like structures in amacrine cells, which failed to elaborate a tau-1-positive axon. In the SB43512-treated Six3:eGFP transgenic fish [31], the eGFP-positive amacrine cells, which normally generate a single axon-like process that extends within the IPL, showed multiple, but normally-oriented processes (39% ± 3% of amacrine cells in Six3:eGFP dissected embryos; n = 100; Fig 3G–3I). Overall, these data show that SB43512 administration causes abnormalities in the IPL and phenocopies the defect in the specification of amacrine axon-like structures observed in the retinas of miR-181a/b morphants. We next investigated the process of RGC axon elongation in SB43512-treated embryos, by taking advantage of the Ath5:eGFP transgenic cell line [32]. 2-D confocal image reconstructions of St32 Ath5:eGFP transgenic whole heads allowed us to visualize the optic nerve routes (dotted white lines in Fig 3K–3M). At St32, SB43512-treated embryos showed a significant reduction of RGC axon growth, compared to control-treated embryos (57% ± 5% of SB43512-treated Ath5:eGFP embryos analyzed; n = 100; Fig 3K–3M). Altogether, these data suggest that the SB43512 treatment causes a failure in optic nerve elongation that recapitulates the RGC defects previously observed in miR-181a/b morphants (Fig 3L and 3M) [14].

**Fig 4:**
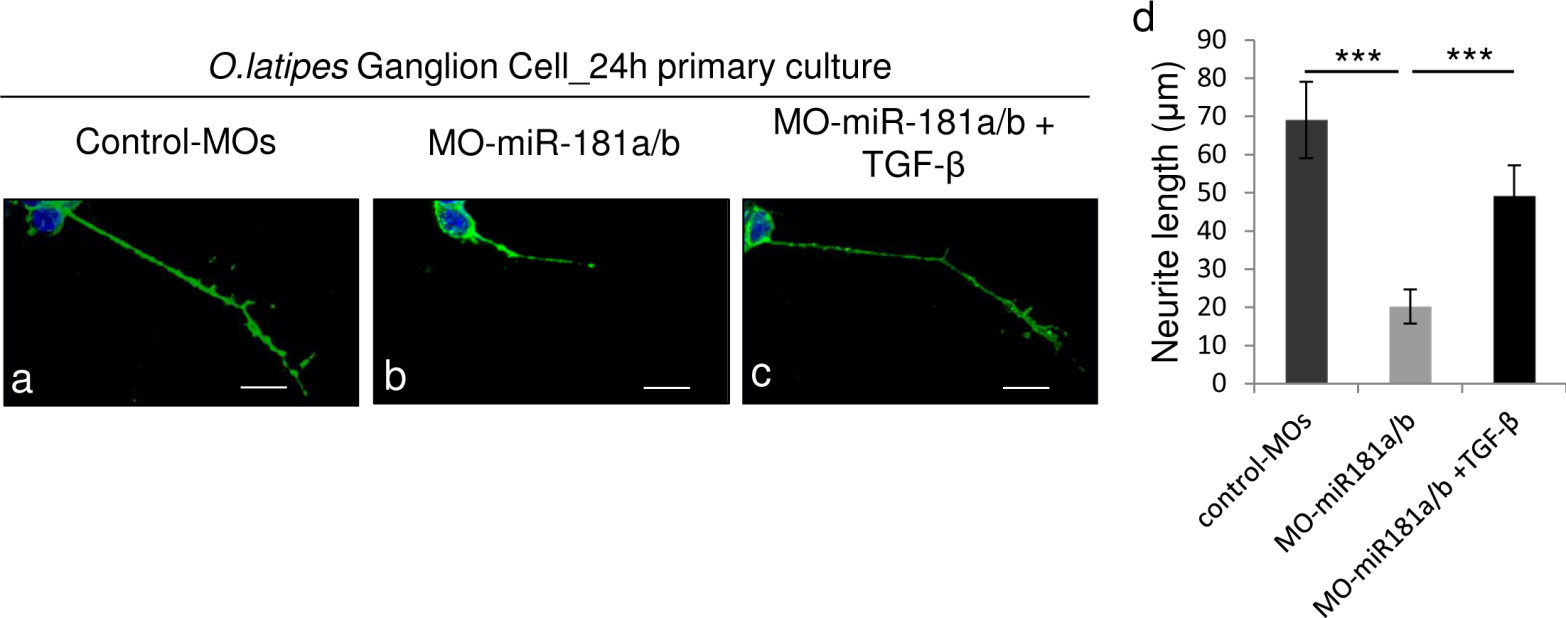
TGF-β administration rescues axon defects in miR-181a/b depleted RGCs. **(a-c)** Representative images from primary RGC cultures from St30 control-MOs (a), miR-181a/b morphant (b) and TGF-β-treated miR-181a/b morphant medaka fish embryos (c). The RGC axon length defect was rescued by treatment with TGF-β (c). Scale bars: 10μm. **(d)** Quantification of RGC axonal length. Data are means +SEM (n = 100) from three independent cell culture experiments. ***P <<0.001 (one-way ANOVA).

**Fig 5:**
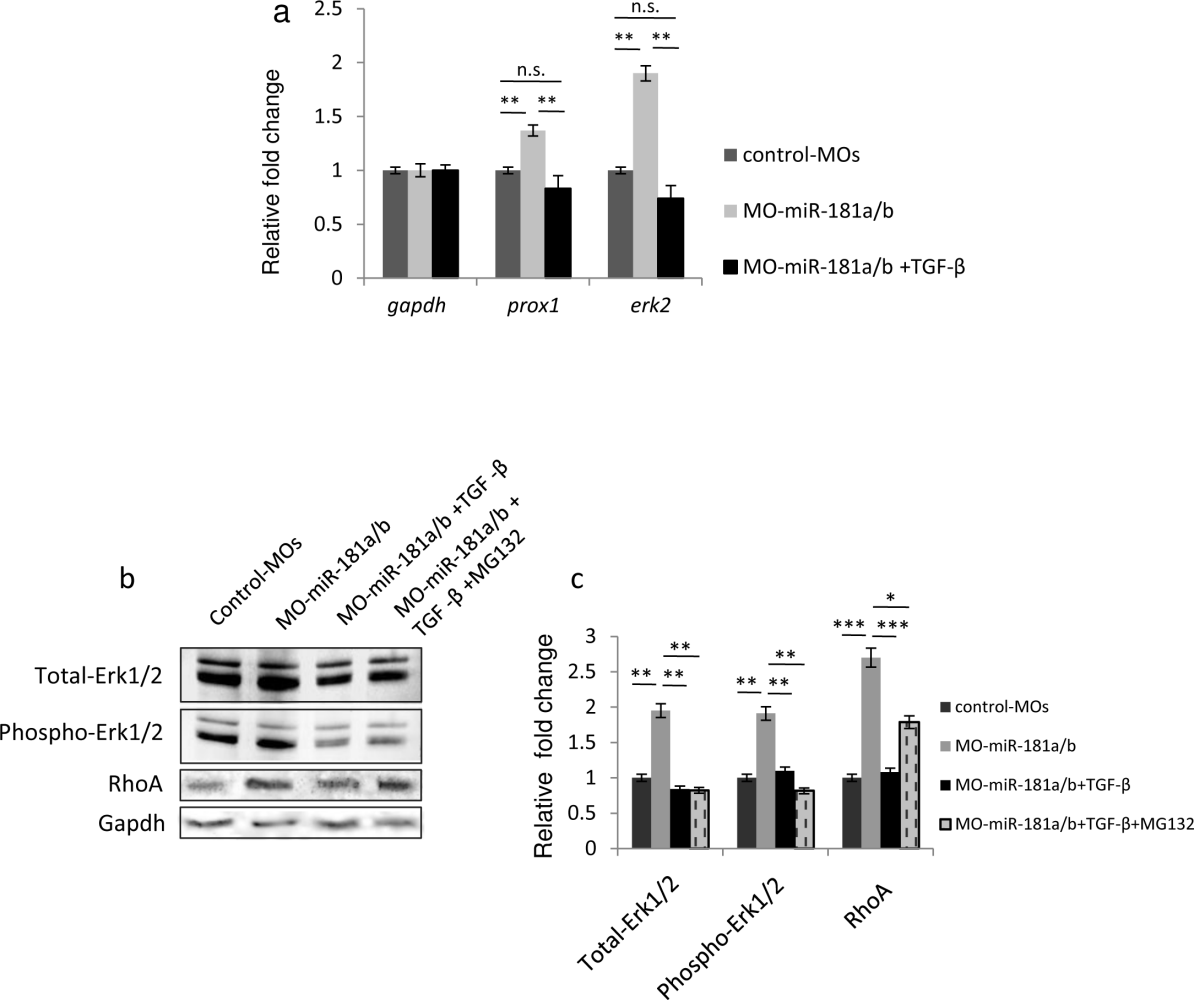
TGF-β signaling regulates RhoA levels via two independent and synergistic cascades. **(a)** qRT-PCR analysis of erk2 transcripts in total eye RNA derived from St32 control-MOs, miR-181a/b morphants and TGF-β-treated miR-181a/b morphants. The TGF-β-mediated increase of miR-181a/b caused a rescue of miR-181a/b target transcripts, such as *prox1* and *erk2*, in miR-181a/b morphants. **(b, c)** Representative Western blotting on protein from St32 eyes (b) and corresponding quantification (c) show that administration of TGF-β to MO-miR-181a/b embryos leads to restoration of total-, phospho-Erk2 and RhoA protein levels. When MO-miR-181a/b embryos were treated with both TGF-β and the proteasomal inhibitor MG132, total- and phospho-Erk2 protein levels were still rescued, whereas RhoA levels were only partially rescued. Data are means +SEM.* P <0.05; **P <0.01; *** P <0.001 (two-way ANOVA).

To further test the possible interplay between TGF-β signaling, miR-181a/b expression and ERK signaling as well as its contribution to retinal development, we treated miR-181a/b morphants with TGF-β. The administration of TGF-β at a concentration of 10ng/ml from St30 onwards did not have any impact on retinal cell death (Fig 1K–1M) and layering in control-treated embryos (Fig 3B). On the other hand, TGF-β-treated miR-181a/b morphants showed a significant rescue of the retina phenotype and of the defects in neuritogenesis (90% ± 2% of embryos; n = 400; Fig 3E, 3F, 3J and 3N). Moreover, we also observed a significant increase in RGC axon growth in TGF-β-treated vs. untreated miR-181a/b morphants (Fig 4A–4D). As previously reported [14], the axons of miR-181a/b morphant RGCs were significantly shorter than those of control MOs in 24-h RGC *in vitro* primary cultures from St30 Ath5:eGFP transgenic embryos. TGF-β administration to *in vitro* primary RGC cultures from miR-181a/b morphants rescued the axon length defects (Fig 4D). This rescue was paralleled by the restoration of miR-181a/b target transcript levels (Fig 5A) and by the rescue of total-, phospho- Erk2 and RhoA protein levels (Fig 5B and 5C). It was recently reported that TGF-β, by acting on the Par6/Smurf1 cascade, is able to regulate RhoA ubiquitination and its consequent degradation in brain neurons, thus inducing correct axon specification [10]. To test if restoration of RhoA levels in TGF-β–treated miR-181a/b morphants is mainly due to RhoA degradation, independently of miR-181a/b and ERK/MAPK signaling, we administered the MG132 compound, a specific proteasomal inhibitor, to TGF-β–treated morphants. In TGF-β/MG132–treated miR-181a/b morphants, we observed an incomplete rescue of RhoA protein levels, compared with the RhoA rescue observed in the TGF-β–treated morphants (Fig 5B and 5C), whereas MG132 had no effects on the TGF-β–mediated rescue of total and phospho-Erk2 protein levels (Fig 5B and 5C). These data demonstrate that TGF-β regulates retinal axon specification by mediating RhoA decrease via two independent but synergistic cascades, i.e., the Par6/Smurf1 cascade and the miR-181/ERK cascade, thus revealing a previously unidentified role for TGF-β in this developmental event.

### TGF-β signaling regulates miR-181a/b action on the *erk2* transcript

**Fig 6:**
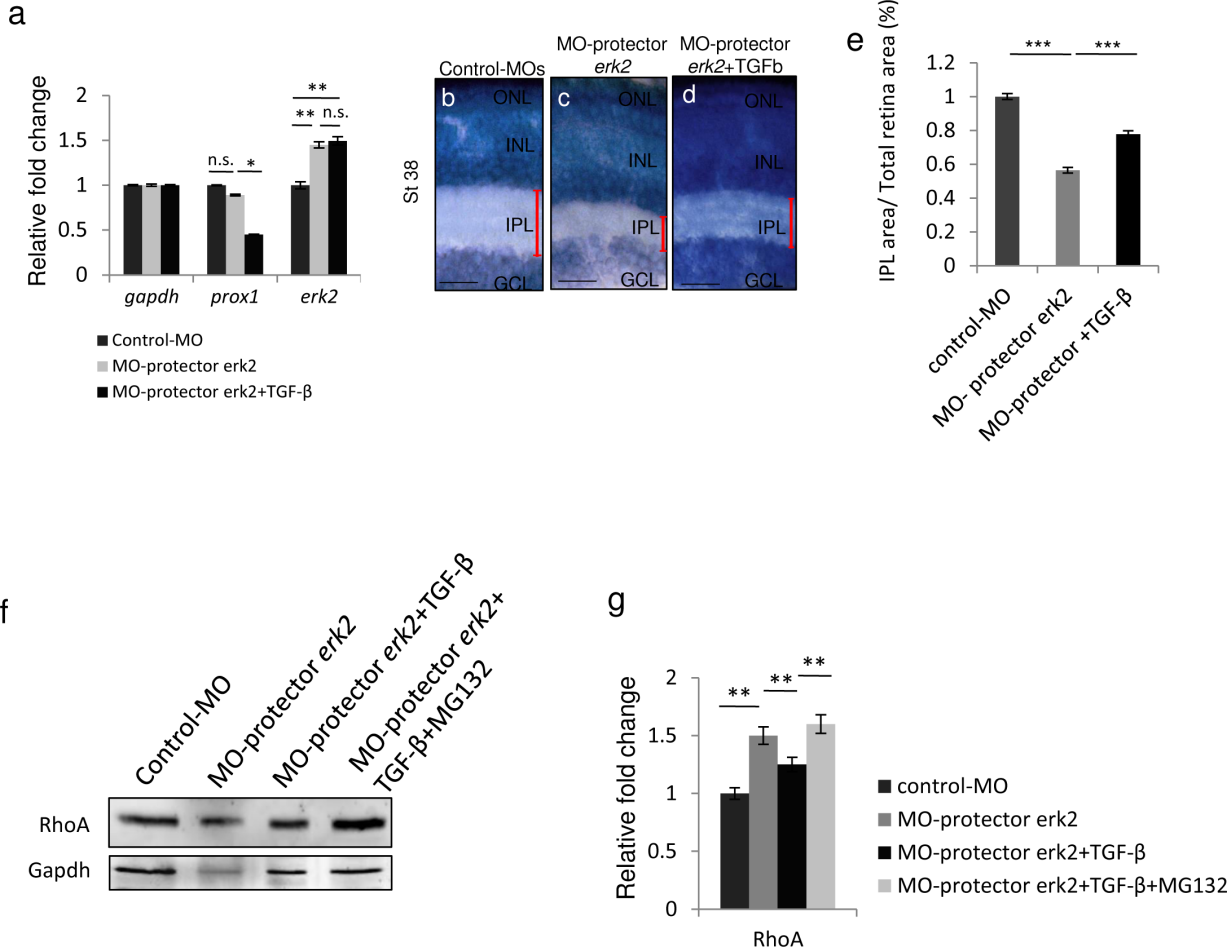
TGF-β signaling regulates *erk2* expression by modulating miR-181a/b levels. **(a)** qRT-PCR analysis of *prox1* and *erk2* transcripts in RNA derived from St32 control-MOs, MO-protector-*erk2*–injected and TGF-β-treated MO-protector-*erk2* medaka fish eyes. The TGF-β rescue on the transcript levels of miR-181a/b targets was mediated by the miR-181a/b increase. Indeed this effect on *erk2* was completely abolished in the MO-protector-*erk2* embryos (a), while other miR-181a/b targets, such as *prox1*, whose miR-181 binding sites are unaffected by the MO-protector, were still sensitive to TGF-β action. Data are means ± SEM. * P <0.05; ** P <0.01 (two-way ANOVA). **(b-d)** Retinal frontal sections of St40 Control (b), MO-protector-*erk2* (c) and TGF-β-treated MO-protector-*erk2* medaka fish embryos (d) processed for Richardson-Romeis staining. Red bars, IPL thickness. Scale bars: 20μm. **(e)** Quantitative analysis of IPL thickness, indicated as the ratio in the central retina between the IPL area and total retinal area. Data are means ± SEM. *** P <0.0001 (one-way ANOVA). **(f-g)** Representative Western blotting on protein from St32 eyes (f) and corresponding quantification (g) show that administration of TGF-β to MO-protector-*erk2* embryos leads to partial rescue of RhoA protein to levels. When MO-protector-*erk2* embryos were treated with both TGF-β and the proteasomal inhibitor MG132, RhoA levels were not rescued anymore. Data are means +SEM. **P <0.05 (one-way ANOVA).

Our data so far demonstrate that TGF-β has an inhibitory effect on *erk2* expression *in vivo*. To confirm that the TGF-β effect on *erk2* transcript levels was indeed mediated by the action of miR-181a/b, we administered TGF-β to *erk2*-morpholino-‘target protector’-injected embryos [33] (MO-protector-*erk2*; see S1 Table). The use of MO-protector-*erk2* allowed us to specifically disrupt the interaction between miR-181a/b and its target site in the 3′-UTR of *erk2* mRNA. The action of MO-protector-*erk2* leads to both an increase of *erk2* transcript levels, with no effect on other miR-181a/b targets such as *prox1* (S1A Fig), and to the increase of both total and phospho-Erk2 protein levels (S1B and S1C Fig). Injection of MO-protector-*erk2* generally phenocopied the miR-181a/b knock-down resulting in IPL defects (S1D–S1G Fig), impaired optic nerve growth (S1H–S1J Fig), and defects in amacrine cell neuritogenesis (S1K–S1M Fig). By quantitative RT-PCR (qRT-PCR) analysis, we observed that *erk2* up-regulation in MO-protector-*erk2*-injected eyes was not rescued by TGF-β treatment (Fig 6A). This indicates that in MO-protector-*erk2* embryos the TGF-β-mediated increase of miR-181a/b does not have an impact on *erk2* transcript levels because of the disrupted interaction between miR-181a/b and its target site in the 3′-UTR of the *erk2* mRNA, while it is still able to act on the levels of other targets such as *prox1* (Fig 6A). These data corroborate the hypothesis that TGF-β has an inhibitory *in-vivo* effect on *erk2* via miR-181a/b.

To further reinforce the importance of the role of TGF-β in regulating *erk*2 during retinal axon specification and growth, we observed that the lack of rescue in *erk2* transcript levels in TGF-β-treated MO-protector-*erk2* embryos was accompanied by a weaker rescue of the defect in IPL thickness (Fig 6B–6E). The latter was associated with a partial restoration of RhoA levels (Fig 6F and 6G), strongly supporting the idea that it could be due to the effect of TGF-β on the Par6/Smurf1 cascade. In hippocampal neurons, TGF-β administration induces the correct axon specification through stimulation of the TGF-βR2 receptor, thus leading to Par6 phosphorylation, which in turn leads to the local recruitment of the ubiquitin ligase Smurf1 and subsequent proteasome-mediated degradation of RhoA [10]. We therefore figured that if TGF-β directly controls levels of RhoA through both TGF-β/Par6/Smurf1-induced degradation and TGF-β/miR-181a/b/MAPK signaling *in vivo*, the administration of the MG132 compound, an inhibitor of proteasomal degradation, to TGF-β–treated MO-protector-*erk2* embryos should prevent the partial rescue of RhoA levels. Indeed, western blot analysis demonstrated that in TGF-β/MG132–treated MO-protector-*erk2* eyes, the partial rescue of RhoA expression levels was absent because of the inhibition of both cascades (Fig 6F and 6G).

**Fig 7:**
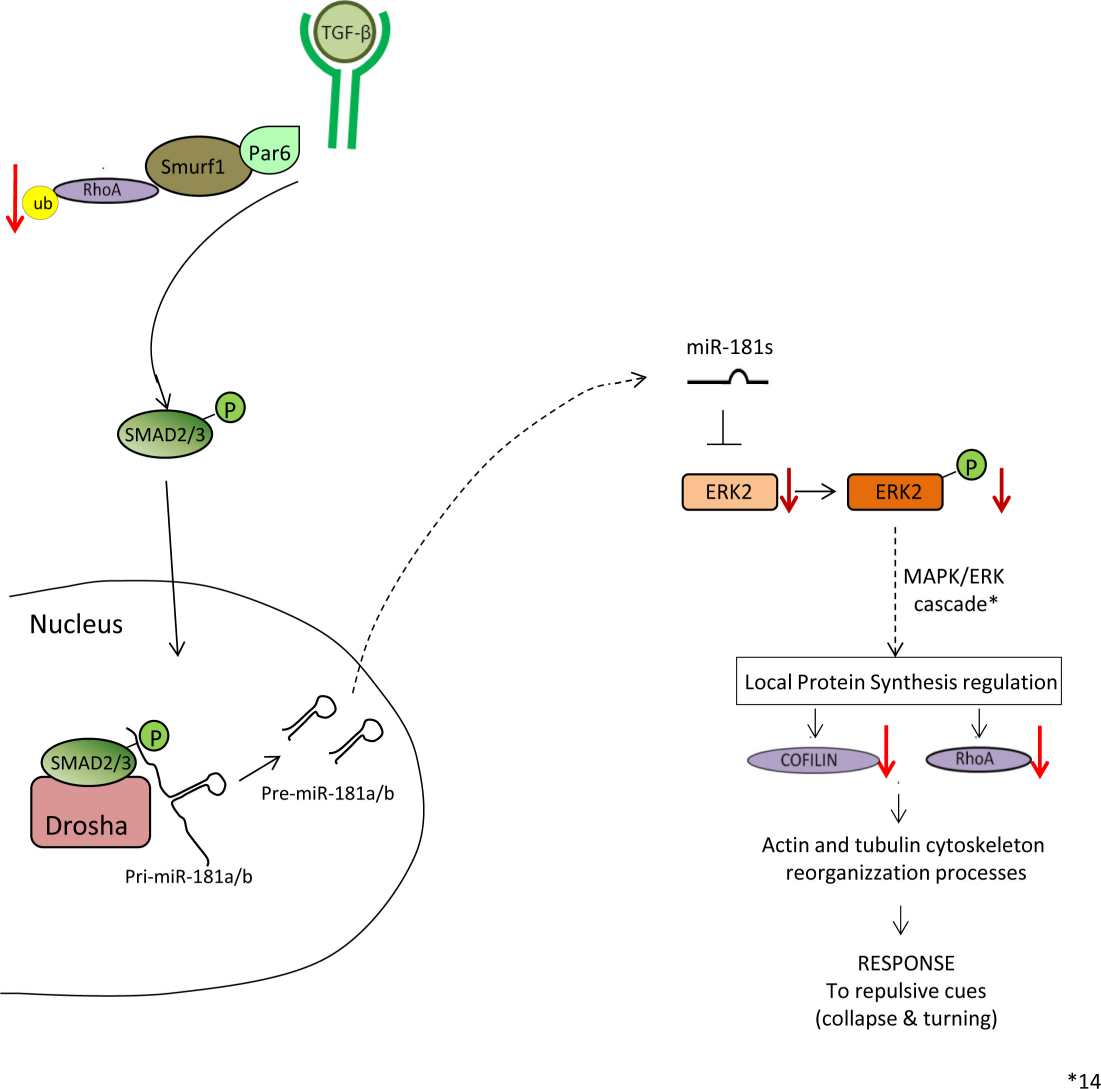
Model of TGF-β cascades in retinal axon specification and growth. The TGF-β pathway regulates axon growth in the retina via two independent and synergistic pathways: the Par6/Smurf1 and the miR-181/ERK pathways. TGF-β–mediated activation of the Par6/Smurf1 cascade leads to ubiquitination and degradation of RhoA. On the other side, TGF-β also generates increased miR-181a/b levels, enhancing the process of miRNA maturation via activation of the SMAD2/3 protein. In turn, by fine modulation of the MAPK/ERK signaling pathway, miR-181a/b has an inhibitory effect on cofilin and RhoA production.

Altogether, these data reveal the existence of a previously unidentified molecular network during retina development, through which the TGF-β pathway antagonizes MAPK/ERK signaling via miR-181a/b action, thereby controlling visual connectivity through two independent and synergistic cascades (Fig 7).

## Discussion

Axon generation in neurons is largely orchestrated by the cooperation between signaling molecules and transcription factors participating to hierarchical molecular networks. Specific components of these networks are “hub” molecules, which act as specific nodes in gene regulation, thereby enhancing the appropriate responsiveness of cells to external and internal stimuli. Intensive research on axon specification, growth and pathfinding in retinal development has led to the identification of most key players [34–36], although many of the regulatory associations between them are still ill defined, thus hampering a detailed understanding of the molecular mechanisms behind these biological processes. Our previous studies have shown that the miRNAs miR-181a/b are, beginning with the initial phases of retinal development, essential for the proper establishment of visual system connectivity and function by acting on MAPK/ERK signaling [14]. The negative modulation exerted by the miR-181/ERK pathway on Cofilin/ADF (Actin Depolarization Factor) and RhoA protein synthesis is necessary for proper neuritogenesis in both amacrine cells and RGCs via local cytoskeletal rearrangement [14].

In this study, we identified the TGF-β pathway as a regulator of miR-181a/b expression during vertebrate retinal development. We show that TGF-β stimulation up-regulates endogenous miR-181a/b expression leading to a rescue in neuritogenesis and axon growth alterations observed in miR-181a/b–depleted amacrine cells and RGCs. Our study provides the first evidence of a role of TGF-β signaling in neuritogenesis in the retina, in concert with the Par6/Smurf1 molecular cascade, previously reported in cortex cells [10]. Our data further support the previously reported role of TGF-β signaling in eye development. Its activity is required during optic cup formation, retinal pigment epithelium (RPE) and neural retina (NR) precursors specification, dorso-ventral patterning of the optic cup, retina proliferation and cell death [2–4] and axon guidance [34]. In this view, our findings demonstrate that TGF-β modulates miR-181a/b expression levels during a period of intense synapse formation in the visual system in order to define proper connectivity.

In previous studies, TGF-β was reported to regulate the expression of miR-181 family members at either the transcription or processing levels, depending on cell type. In hepatocarcinogenesis, the TGF-β effector SMAD4 appears to control the transcription of miR-181b [15], whereas TGF-β induces miR-181a/b at the post-transcriptional level through SMAD2/3-dependent miRNA maturation in breast cancer [16]. Our study shows, for the first time *in vivo*, that TGF-β regulates miR-181a/b at the post-transcriptional level in the retina. This regulation is likely to be mediated by a SMAD2/3-dependent mechanism since specific inhibition of SMAD2/3 phosphorylation, obtained via SB43152 treatment [21], leads to a significant reduction in mature miR-181a and miR-181b levels in the retina.

This study also highlights miR-181a/b as crucial molecular hubs between the TGF-β and MAPK/ERK signaling pathways (Fig 7) during retinal development. Recently, our studies demonstrated that miR-181a/b act as key regulators of retinal axon specification and growth through negative modulation of MAPK/ERK signaling [14]. Here we show a specific TGF-β-mediated regulation of MAPK/ERK signaling pathways through modulation of miR-181a/b levels. Obviously, we expect additional molecules to be part of the molecular network controlled by miR-181a/b to ensure proper retinal neuritogenesis. Interestingly, Neuropilin-1 (Nrp1), a direct target of miR-181b [36], appears to bias the polarized extension of RGC dendrites, by acting as a mediator of Semaphorin signaling during IPL formation [37]. Since Nrp1 can act as a co-receptor for TGF-β in cancer cells [38], it would be interesting to determine whether the TGF-β–mediated miR-181a/b regulation we observe in the retina also involves Nrp-1 regulation.

Despite the established role of TGF-β/BMP superfamily members in promoting axon specification and neurite outgrowth [10, 18, 26–28], a lot remains to be unraveled on the link between the TGF-β and MAPK/ERK signaling pathways in these processes. On one hand, it was previously reported that in rat sympathetic neurons, either TGF-β/BMP administration or MAPK/ERK inhibition enhance neurite growth [27], whereas, on the other hand, TGF-β treatment of RGCs (RGC-5), although enhancing neurite outgrowth, led to only a minimal decrease in ERK1/2 activation [28]. Our results contribute to sharpen these partially controversial findings [27, 28]. By applying an integrated strategy involving both *in vivo* and *in vitro* approaches, we have identified a link between the TGF-β and MAPK/ERK signaling pathways, at least in retinal cells. It is possible that the lack of ERK1/2 activation, previously observed in RGC-5 cells, was due to a lower concentration of TGF-β administered [28]. Our data demonstrate that TGF-β has a role in the negative regulation of MAPK/ERK signaling, by acting on *erk2* transcript via miR-181a/b. It is tempting to speculate that the molecular network in which miR-181a/b participate might also be relevant for other events in which TGF-β signaling and the MAPK/ERK pathway have antagonistic roles [39]. In mouse neurons, TGF-β signaling specifies axons and promotes their growth [10]; conversely, its deficiency results in axon, dendrite and age-related degeneration [11]. The activation of the MAPK/ERK pathway instead inhibits neurite growth and is involved in the pathogenesis of Parkinson’s disease [40]. Our discovery of a TGF-β-mediated inhibition of the MAPK/ERK pathway via miR-181a/b will provide further ground to better elucidate the functional interactions between TGF-β and MAPK/ERK signaling under physiological and pathological conditions of the CNS, as miR-181a/b are predominantly expressed in this tissue.

In conclusion, our results provide a better understanding of the signaling network underlying visual system development and function. We report that the miR-181/ERK regulatory network is under the control of TGF-β signaling and works in concert with the TGF-β/Par6/Smurf1 cascade in retinal axon specification. Our findings provide novel information on how visual system connectivity and function are originated during eye development and reinforce the role of miRNAs in finely modulating interactions between members of different signaling families in fundamental biological processes.

## Materials and Methods

### Medaka fish stocks

#### Ethics statement

All studies on fish were conducted in strict accordance with the institutional guidelines for animal research and approved by the Italian Ministry of Health; Department of Public Health, Animal Health, Nutrition and Food Safety in accordance to the law on animal experimentation (article 7; D.L. 116/92; protocol number: 00001/11/IGB; approval date June 6, 2011). Tricaine methanesulfonate (MS-222; Sigma Aldrich) was used for ‎euthanasia (200-300mg/L) and anesthesia (0,05%) [41]. Furthermore, all animal treatments were reviewed and approved in advance by the Ethics Committee of the Institute of Genetics and Biophysics, IGB Animal House, (Naples, Italy).

Samples of the Cab strain of wild-type medaka fish were kept and staged as described previously [42]. Staging of retinal development in medaka fish was determined in accordance to Kitambi et al. [43].

### Morpholino injections

All MOs (Gene Tools, LLC) were injected into fertilized one-cell embryos, at the concentration reported in S1 Table. The optimal MO concentrations (see S1 Table) were determined on the basis of morphological criteria. The specificity and inhibitory efficiency of each MO were determined as described previously [14, 44] (see S1 Supporting Text).

### TUNEL assay

Control, MO-*tgfβr1*, SB43152- and TGF-β-treated Control, MO-miR181a/b and TGF-β-treated MO-miR-181a/b embryos were subjected to anesthesia before fixation at the stage of interest (St32) by an overnight incubation in 4% paraformaldehyde in PBS-Tween (PTW) at 4°C and then stored at -20°C in methanol. After rehydration, whole mount TUNEL assay was performed using the In Situ Cell Death Detection Kit (Roche, Mannheim, Germany) according to the manufacturer’s instructions.

### Richardson-Romeis staining (histo-blue sections)

The animals were subjected to anesthesia before fixation at the stage of interest (St40) by an overnight incubation in 4% paraformaldehyde in PBS-Tween (PTW) at 4°C, and then incubated overnight in 15% sucrose/PTW at 4°C, and then again incubated overnight in 30% sucrose/PTW at 4°C. Cryosections of the embryos were processed for the Richardson-Romeis staining. The Richardson-Romeis solution (1% Azur II solution, 1% methylene blue in 1% borax; 1:1) was applied briefly on slides on a heater (60°C). After the removal of the solution, the sections were washed briefly with tap water. The sections were left over night in water, dried on a heater, and closed with phosphate-buffered saline (PBS)/50% glycerol. A number of 100 St40 animals were analyzed for each treatment.

### Transgenic lines

The Ath5:eGFP [32] and Six3.2:eGFP [31] transgenic lines were used to analyze the amacrine cells and RGCs. Six3.2:eGFP transgenic embryos were injected either with control-MO, or MO-miR-181a/b, or MO-protector-*erk2*, or treated with SB43152, or TGF-β or DMSO. The embryos were subjected to anesthesia before being fixed at the stage of interest (St40) by an overnight incubation in 4% paraformaldehyde in PBS-Tween (PTW) at 4°C, and then incubated overnight in 15% sucrose/PTW at 4°C, and then again incubated overnight in 30% sucrose/PTW at 4°C. Cryosections of the control and morphant transgenic embryos were washed three times with PTW and were counterstained with DAPI (Vector Laboratories). The slides were photographed under LSM710 Zeiss confocal microscopy. For amacrine cell analysis, a number of 100 Six3.2:GFP animals for each treatment were analyzed in histological sections at St40. Ath5:eGFP transgenic embryos were injected either with control-MO, or MO-miR-181a/b, or MO-protector-*erk2*, or treated with SB43152, or TGF-β or DMSO. For optic nerve analysis using 2-D confocal images reconstruction (see below), the embryos were then fixed at the stage of interest (St32) by an overnight incubation in 4% paraformaldehyde in PBS-Tween (PTW) at 4°C. A number of 100 animals for each treatment were analyzed at St32. For axon length analysis using the *in vitro* primary culture of RGCs, the eyes from Ath5:eGFP embryos were collected at St30 (see protocol below).

### 2-D reconstruction of confocal images of St32 Ath5:eGFP transgenic whole-heads

Control-MOs or DMSO-treated, miR-181a/b morphant, SB432542-treated, TGF-β-treated miR-181a/b morphant, MO-protector-*erk2* injected Ath5:eGFP transgenic embryos were subjected to anesthesia before fixation at St32 by an overnight incubation in 4% paraformaldehyde in PBS-Tween (PTW) at 4°C. Fixed embryos were washed in PTW and then included in low-melting agarose in 35mm x 10mm petri-dish. Leica TCS SPE confocal Laser Scanning Microscope was used for the acquisition. Confocal z-stack images of the embryos’ whole heads were acquired by spanning a range of 100μm (1μm each step) for each head and by ensuring that all the GFP signals were included in the z-stack. The 2-D reconstructions were obtained using the Maximum Projection tool (LAS-AF software 2011.4.0) that allowed to visualize the accumulation of GFP signals derived from every single plane on a single image.

### RNA extractions

Total RNA was obtained from a pool of St32 eyes of control, control treated with SB43152 or TGF-β, miR-181a/b morphant, TGF-β-treated miR-181a/b morphant, MO-protector-*erk2* and TGF-β-treated MO-protector-*erk2* medaka fish embryos. Each pool of eyes contained at least 80–100 eyes. Egg envelopment was removed and embryos from each treatment were subjected to anesthesia, eyes were dissected and immediately processed with a pestle in QIAzol Lysis Reagent (Qiagen). The RNAs were extracted using RNeasy extraction kits (Qiagen), according to the manufacturer’s instructions. During the extraction protocol the RNAs were digested with DNaseI (Qiagen) to remove contaminating genomic DNA, according to the manufacturer’s instructions. The extracted RNAs were quantified using NanoDrop2000 (Thermo Scientific) and their integrity and purity (A260/A280) were assessed using RNAscreen TapeStation 2200 System (Agilent Technologies), according to the manufacturer’s instructions (see S2 Table for data obtained from representative RNA sample from each treatment). The RNA samples were stored at -80°C.

### Quantitative Real-Time PCR

For quantitative Real-Time PCR (qRT-PCR) experiments, cDNAs were generated using the QuantiTect Reverse Transcription Kit (Qiagen), according to the manufacturer’s instructions. For each cDNA preparation, 1000ng from each RNA sample was used in a final reaction volume of 20μL. By using the RT Primer Mix (QuantiTect Reverse Transcription Kit; Qiagen), a specially optimized mix of oligo-dT and random primers, it is possible to obtain cDNA synthesis from all regions of transcripts, even from 5' regions. During the retro-transcription protocol purified RNA is briefly incubated in gDNA Wipeout Buffer to effectively remove contaminating genomic DNA that could persist in RNA samples even after DNaseI digestion. The Quantiscript Reverse Transcriptase enables the retro-transcription of RNA at low temperatures. The entire reaction takes place at 42°C and the Quantiscript Reverse Transcriptase is then inactivated at 95°C. cDNAs were stored at -20°C.

The primers for qRT-PCR reactions were designed using *in silico* tools that allowed to predict their melting temperature (*T*_m_) and to avoid the possibility of self-annealing or primer dimerization (www.basic.northwestern.edu/biotools/oligocalc.html). The *in silico* specificity of the designed primers was tested using the BLAST/BLAT tool in Genome Browser (https://genome.ucsc.edu/) or ensembl (http://www.ensembl.org/index.html). No homology to pseudogenes, alternative spliced forms or unexpected targets were recognized by the designed primers in the *in silico* test. The primers were designed spanning on two different exons to avoid genomic DNA amplification. The primers specificity was empirically evaluated using melt-curve and electrophoresis gel analysis (S2 Fig) and their efficiencies were empirically validated in qRT-PCR reactions using a target-specific standard dilution series carried out with the Roche Light Cycler 480 system. In S3 Table are reported the primer sequences, amplicon location and size, the assay performance characteristics such as efficiency, slope and r^2^ obtained for each primer pair, as suggested by Bustin et al. [45]. For each couple of primers: Ct>40 was observed for the No-reverse Transcription Controls (NTCs), LOD Ct <35 and *y* intercept: 35–40. Every qRT-PCR reaction was performed using 1μL of synthesized cDNA in each tube in a final volume of 20μL. Each primer was used at a final concentration of 0.41μM. The mean Ct values +/- SEM for all genes analyzed in each qRT-PCR experiment are reported in S4 Table. The thermocycling conditions are reported in S5 Table. The quantification data, obtained in qRT-PCR reactions on cDNAs from the different treatments, are expressed in terms of cycle threshold (Ct). The *hprt* and *gapdh* genes were used as endogenous controls for the experiments, their utility as reference genes was assessed previously [14, 46–48]. The *prox1* gene was used as positive control, as it is an already validated target for miR-181 [29]. The Ct values were averaged for each in-plate technical triplicate. The averaged Ct was normalized as difference in Ct values (ΔCt) between the mRNA in analysis and each reference gene in each sample in analysis. Then the ΔCt values of each sample were normalized with respect to the ΔCt values of the control (ΔΔCt). The variation was reported as fold change (2^-ΔΔCt^). Once verified that the observed mRNA variations were not influenced by the use of *hprt* or *gapdh* as reference gene, we reported in the graphs the data normalized using *gapdh* as reference gene. Each plate was performed in duplicate and all the results are shown as means ± SEM of three independent biological assays.

### Mature miRNA quantitative assay

For the mature miRNA quantitative assay we used the hydrolysis probes (TaqMan, Applied Biosystem) for the detection of mature miR-181a and mature miR-181b sequences. TaqMan MicroRNA Assays probes are highly specific and quantitate only mature miRNAs, not precursors. The cDNAs for mature miRNAs analysis were generated using the TaqMan MicroRNA Reverse Transcription Kit with miRNA-specific primers, according to the manufacturer’s instructions. The thermocycling conditions are reported in S6 Table. The quantification data, obtained in TaqMan-PCR reactions on TaqMan-cDNAs from the different treatments, are expressed in terms of the cycle threshold (Ct). A custom TaqMan probe designed on the medaka fish U6 snoRNA was used as endogenous control for the experiments. The Ct values were averaged for each in-plate technical triplicate. The averaged Ct was normalized as difference in Ct values (ΔCt) between the miRNA in analysis and U6 snoRNA in each sample in analysis. Then the ΔCt values of each sample were normalized with respect to the ΔCt values of the control (ΔΔCt). The variation was reported as fold change (2^-ΔΔCt^). Each plate was performed in duplicate and all the results are shown as means ± SEM of three independent biological replicates.

### Statistical analysis

A paired T-Test was carried out to test for variation of a single factor between two variables (two samples/treatments). A one-way ANOVA with Tukey HSD (Post-Hoc test) was carried out to test for variation of a single factor among multiple variables (multiple samples/treatments), while a two-way ANOVA with Tukey HSD (Post-Hoc test) was performed to test for variation of more than one factor among multiple variables (multiple samples/treatments).

### Protein isolation and Western blotting

Total protein extracts from a pool of St32 eyes were obtained from control, control treated with SB43152, miR-181a/b morphant, miR-181a/b morphant treated with TGF-β, or TGF-β plus MG132, MO-protector-*erk2* and MO-protector-*erk2* treated with TGF-β, or TGF-β plus MG132 embryos. Each pool of eyes contained at least 80–100 eyes. The eggs envelopment was removed and embryos from each treatment were subjected to anesthesia, the eyes were dissected and immediately processed with a pestle in RIPA buffer (50mM Tris-HCl, 1 mM EDTA, 150mM NaCl, 1% Triton-100X, 0.1%SDS, protease inhibitor cocktail tablet [Roche]). The protein extract concentrations were determined using the Bio-Rad protein assay (Bio-Rad, Munich, Germany). A total of 20μg to 40μg of proteins from each sample was loaded on 12% or 15% SDS-polyacrylamide gels. For western blotting, the gels were electroblotted onto nitrocellulose filters and sequentially immunostained with the primary antibodies (see S7 Table) overnight at 4°C, and then with peroxidase-labeled secondary antibodies (GE Healthcare, Little Chalfont Buckinghamshire, UK) at room temperature for 1–2 h. The Western blotting was revealed using the Pierce ECL Western blotting substrate (Thermo Scientific) and the images were acquired using the Chemidoc-IT UVP and the Visionworks Software. The Western blotting data were quantified using the ImageJ analysis package (National Institutes for Health). The signals for each protein staining were quantified and then normalized for the Gapdh in the same sample (internal normalization). These normalized values were then compared to the values in the control sample. The average of the normalized values from three different biological replicates is illustrated as relative fold change.

### Primary culture of medaka fish retinal cells

For the generation of *in-vitro* primary cultures of RGCs, we used the Ath5:eGFP transgenic medaka fish line, in which GFP is expressed in all of the RGCs. The embryos were subjected to anesthesia. The eyes extracted from medaka fish Ath5:eGFP control and morphant embryos at St30 (around the onset of RGCs differentiation) were dissociated in 100μL L15 medium supplemented with 10% fetal bovine serum, 100U/ml penicillin and 50mg/ml streptomycin, 20μl 10mg/ml Trypsin (in PBS) and incubated at 37°C (shaken periodically). After the addition of 20μl soya bean trypsin inhibitor (20mg/ml in PBS), mechanical dissociation was obtained using a syringe with a G27 needle. The cells were seeded onto 13mm coverslip-bottomed dishes covered with 20μg/ml poly-D-lysine (bidistilled water) and 10μg/ml laminin (in PBS), in 600μl complete L15 + 20μl N2 supplement medium (100×), pre-heated at 37°C. The cells were then kept at 30°C for 24 h. Once fixed in 4% paraformaldehyde in PBS-Tween (PTW), the cells were washed three times with PTW and were counterstained with DAPI (Vector Laboratories). The cells were photographed under LSM710 Zeiss confocal microscopy. The axon length was measured using the ImageJ analysis package (National Institutes for Health). A total number of 100 cells were analyzed from three independent cell culture experiments and the data are represented as means +SEM.

### Drug treatments

The egg envelope was removed with the hatching enzyme. St30 morphant or control embryos were grown in 80μM SB43152, or 10ng/ml TGF-β, or 10ng/ml TGF-β plus 100μM MG132, diluted in 3%DMSO, 1×Yamamoto, for a minimum of 24 h (until St32) to a maximum of 6 days (until St40). For the control experiments, the St30 morphant or control embryos were grown in 1×Yamamoto/3%DMSO.

## Supporting Information

S1 Fig Injection of MO-protector-ERK2 phenocopied miR-181a/b-morphant retinal defects. **(A)** qRT-PCR on total RNA from stage 32 control and Mo-protector-ERK2 injected eyes, for *Erk2* and *Prox1* transcripts, normalized to *GAPDH* transcript levels. *Erk2* levels were increased in MO-protector-ERK2 eyes. Instead *Prox1* transcript levels are not significantly altered, indicating that the MO-protector-ERK2 is specific for the miR-181a/b seed region in the *olErk2* 3’UTR. Data are means ±SEM **, P <0.01 (t-test). **(B-C)** Representative Western blotting (B) and its quantification (C) show increased total and phosphorylated ERK2 protein levels in stage 32 MO-protector-ERK2 eyes, compared with control medaka fish eyes. Data are means ±SEM **, P <0.01 (t-test). **(D-G)** Representative retinal frontal sections of St38 control-MOs (D), miR-181a/b morphant (E), MO-protector-ERK2–injected (F) embryos processed for Richardson Romeis staining. Red bars, inner plexiform layer (IPL) thickness. Scale bars: 20 μm. **(G)** Quantitative analysis of IPL thickness, as the ratio in the central retina between the IPL area and total retinal area. Data are means ±SEM; ***, P <<0.001 (one-way ANOVA). Inhibition of miR-181a/b binding to the *Erk2* target site via MO-protector-ERK2 resulted in decreased IPL thickness, compared to controls. **(H-J)** Representative 2-D reconstruction of confocal images of stage 32 control (H), miR-181a/b morphant (I), MO-protector-ERK2–injected (J) Ath5:GFP transgenic whole-heads. Dotted white lines, optic nerve routes. Injection of MO-protector-ERK2 (J) phenocopied the miR-181a/b-morphant optic nerve length decrease (I). Scale bars: 50 μm. OT, optic tectum. **(K-M)** Representative images of amacrine cells from St38 retinal sections of control-MOs (K), miR-181a/b morphant (L), MO-protector-ERK2–injected (M) Six3:eGFP transgenic embryos. Cell nuclei are stained with DAPI (blue). GFP (green signal) stains amacrine cell soma and neurites; red arrows, Six3 axon-like structure of amacrine cells; red bars, IPL thickess. The amacrine cells of Six3.2 MO-protector-ERK2 phenocopied neuritogenesis defects of the miR-181a/b-morphants. Scale bars: 20 μm. ONL, outer nuclear layer; INL, inner nuclear layer; GCL, ganglion cell layer.
(TIF)

S2 Fig Electrophoresis gel of PCR amplicon for transcripts analysed by qRT-PCR: (A, B). The PCR procucts obtained using the qRT-PCR primers for *gapdh*, *hprt*, *erk2*, *prox1* **(A)** and using the qRT-PCR primers for the pri-miR-181 family members **(B)** were analysed by gel-electrophoresis. The gel-electrophoresis analysis showed the absence of unintended amplification products and no PCR contamination problems. The expected amplicon size, predicted by *in silico* analysis, was confirmed for each PCR product.
(TIF)

S1 Supporting Text Controls for MO injections. (PDF)

S1 Table Sequences of used Morpholinos (MOs) and Oligonucleotides primers. (PDF)

S2 Table Representative RNA Sample characteristics analyzed using RNAscreen TapeStation 2200 System (Agilent Technology). (PDF)

S3 Table Primer sequences and performance characteristics in target specific assay. (PDF)

S4 Table Mean Ct values +/- SEM for all genes analyzed in each qRT-PCR experiment. (PDF)

S5 Table qRT-PCR thermocycling conditions. (PDF)

S6 Table Mature miRNA quantitative assay thermocycling conditions. (PDF)

S7 Table Western blotting conditions used for each antibody. (PDF)
