# Supplementary figures and images for: TGF-β Controls miR-181/ERK Regulatory Network during Retinal Axon Specification and Growth
Source: PLoS One. 2015 Dec 7;10(12):e0144129. doi: 10.1371/journal.pone.0144129 (PMC4671616; doi:10.1371/journal.pone.0144129)

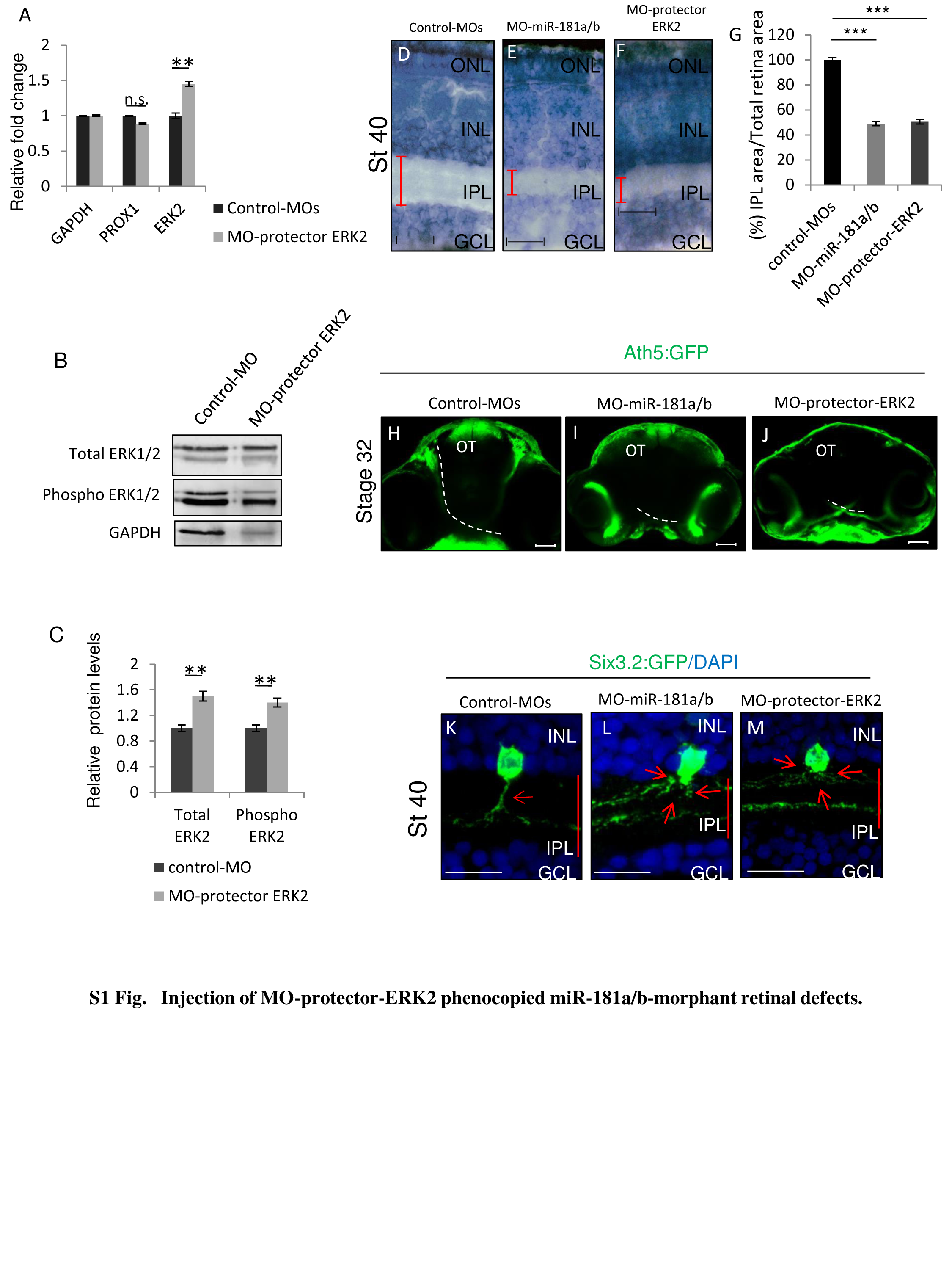

Supplement: S1 Fig — (A) qRT-PCR on total RNA from stage 32 control and Mo-protector-ERK2 injected eyes, for Erk2 and Prox1 transcripts, normalized to GAPDH transcript levels. Erk2 levels were increased in MO-protector-ERK2 eyes. Instead Prox1 transcript levels are not significantly altered, indicating that the MO-protector-ERK2 is specific for the miR-181a/b seed region in the olErk2 3’UTR. Data are means ±SEM **, P <0.01 (t-test). (B-C) Representative Western blotting (B) and its quantification (C) show increased total and phosphorylated ERK2 protein levels in stage 32 MO-protector-ERK2 eyes, compared with control medaka fish eyes. Data are means ±SEM **, P <0.01 (t-test). (D-G) Representative retinal frontal sections of St38 control-MOs (D), miR-181a/b morphant (E), MO-protector-ERK2–injected (F) embryos processed for Richardson Romeis staining. Red bars, inner plexiform layer (IPL) thickness. Scale bars: 20 μm. (G) Quantitative analysis of IPL thickness, as the ratio in the central retina between the IPL area and total retinal area. Data are means ±SEM; ***, P <<0.001 (one-way ANOVA). Inhibition of miR-181a/b binding to the Erk2 target site via MO-protector-ERK2 resulted in decreased IPL thickness, compared to controls. (H-J) Representative 2-D reconstruction of confocal images of stage 32 control (H), miR-181a/b morphant (I), MO-protector-ERK2–injected (J) Ath5:GFP transgenic whole-heads. Dotted white lines, optic nerve routes. Injection of MO-protector-ERK2 (J) phenocopied the miR-181a/b-morphant optic nerve length decrease (I). Scale bars: 50 μm. OT, optic tectum. (K-M) Representative images of amacrine cells from St38 retinal sections of control-MOs (K), miR-181a/b morphant (L), MO-protector-ERK2–injected (M) Six3:eGFP transgenic embryos. Cell nuclei are stained with DAPI (blue). GFP (green signal) stains amacrine cell soma and neurites; red arrows, Six3 axon-like structure of amacrine cells; red bars, IPL thickess. The amacrine cells of Six3.2 MO-protector-ERK2 phenocopied neur [file pone.0144129.s001.tif]

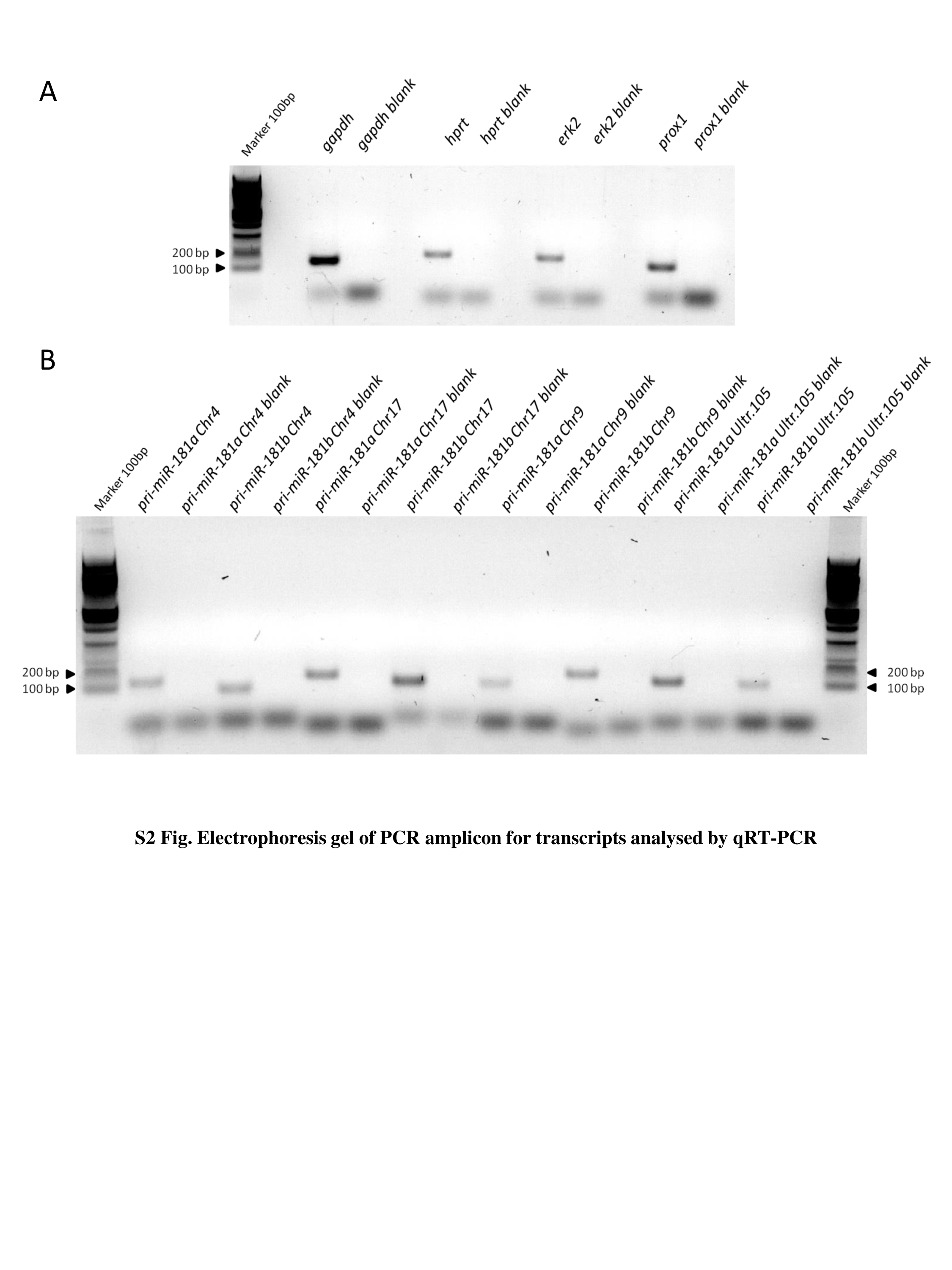

Supplement: S2 Fig — The PCR procucts obtained using the qRT-PCR primers for gapdh, hprt, erk2, prox1 (A) and using the qRT-PCR primers for the pri-miR-181 family members (B) were analysed by gel-electrophoresis. The gel-electrophoresis analysis showed the absence of unintended amplification products and no PCR contamination problems. The expected amplicon size, predicted by in silico analysis, was confirmed for each PCR product. (TIF) [file pone.0144129.s002.tif]
