# Supplementary material for: TGF-β Controls miR-181/ERK Regulatory Network during Retinal Axon Specification and Growth
Source: PLoS One. 2015 Dec 7;10(12):e0144129. doi: 10.1371/journal.pone.0144129 (PMC4671616; doi:10.1371/journal.pone.0144129)
Supplement: S1 Supporting Text — (PDF) [file pone.0144129.s003.pdf]

S1 Supporting Text: Controls for MO injections

MO-TGFβR1 was designed to sterically block the fourth splice donor site of Ol.*TGFβR1* transcript (Fig. S1a). The blocking efficiencies of the MO-TGFβR1 were verified by PCR using a pair of oligonucleotide primers (Ol.*tgfβr1*.ex3F-5'GAGCTGATTCCACGAGACCG3'; Ol.*tgfβr1*.ex5R-5'CTTCACCGCAACCTCCTCGC3') designed on the flanking exons (exon3 and exon5, Fig. S1a). PCR analysis revealed that the MO-TGFβR1 caused a partial retention of an intron as shown by the presence of a higher band amplified in MO-TGFβR1 injected embryos with respect to controls (Fig. S1a).

Activation of p53 is an occasional off-targeting effect of knockdown strategies that induce neuronal p53-dependent cell death accompanied by abnormal tissue development, such as microcephaly, microphthalmia, and others[1]. In this case, the phenotype can be rescued by co-injection of a MO against p53[2]. We sought to exclude that the phenotype in TGFβR1 morphants was related to non-specific neural cell death in response to the injection of our MO. Therefore, we co-injected MO-TGFβR1 with an MO against p53 (MO-p53)[3]. Of note, there were no modifications of the morphant phenotype (data not shown), which confirmed the specificity of the MO-TGFβR1 targeting and the absence of the occasional off-targeting effect due to the knock-down strategy.

1. Robu, M. E. *et al.* p53 activation by knockdown technologies. *PLoS Genetics* 3, e78, doi:10.1371/journal.pgen.0030078 (2007).

2. Eisen, J. S. & Smith, J. C. Controlling morpholino experiments: don't stop making antisense. *Development* 135, 1735-1743, doi:10.1242/dev.001115 (2008).

3. Conte, I. *et al.* miR-204 is required for lens and retinal development via Meis2 targeting. *Proc Natl Acad Sci U S A* 107, 15491-15496, doi:0914785107 [pii].
