# Supplementary material for: TGF-β Controls miR-181/ERK Regulatory Network during Retinal Axon Specification and Growth
Source: PLoS One. 2015 Dec 7;10(12):e0144129. doi: 10.1371/journal.pone.0144129 (PMC4671616; doi:10.1371/journal.pone.0144129)
Supplement: S1 Table — (PDF) [file pone.0144129.s004.pdf]

S1 Table: Sequences of used Morpholinos (MOs) and Oligonucleotides primers

MOs	Sequence	Concentration (mM)
MO-*tgfβr1*	5′-GTTTGGCATGAGGAGTACCTGATCC-3’	0.4
MO-protector-*erk2*	5′-GAACATTCATCTGACCAGAAAAGAT-3’	0.15
MO-miR-181a*	5′-AACTCACCGACAGCGTTGAATGTTC-3’	0.12
MO-miR-181b*	5′-AACCCACCGACAGCAATGAATGTTG-3’	0.12
mm-MO-miR-181*	5′-AAGTCAGCGACACCGTTCAATCTTC-3’	0.12
mm-MO-miR-181b*	5′-AAGCCACGGACACCAATCAATCTTG-3’	0.12
MO-ol*p53*	5′-CGGGAATCGCACCGACAACAATACG-3’	0.09

*Carrella et al. 2015
