# Supplementary material for: TGF-β Controls miR-181/ERK Regulatory Network during Retinal Axon Specification and Growth
Source: PLoS One. 2015 Dec 7;10(12):e0144129. doi: 10.1371/journal.pone.0144129 (PMC4671616; doi:10.1371/journal.pone.0144129)
Supplement: S2 Table — (PDF) [file pone.0144129.s005.pdf]

S2 Table: Representative RNA Sample characteristics analyzed using RNAscreen TapeStation 2200 System (Agilent Technologies)

**RNA Sample**	**Purity (A260/A280)**	**RIN^e**
Control eyes st32	2.08	7.6
Control+TGFβ eyes st32	2.09	6.7
Control+ActD+TGFβ eyes st32	2.07	8.3
MO-miR-181a/b eyes st32	2.03	8.4
MO-miR-181a/b+TGFβ eyes st32	2.01	7.7
MO-protector *erk2* eyes st32	2.08	8.2
MO-protector *erk2*+TGFβ eyes st32	2.1	8.6
Control+ SB43152 eyes st32	2.1	8.6
MO-*tgfβr1* eyes st32	2.02	7.9
