# Supplementary material for: TGF-β Controls miR-181/ERK Regulatory Network during Retinal Axon Specification and Growth
Source: PLoS One. 2015 Dec 7;10(12):e0144129. doi: 10.1371/journal.pone.0144129 (PMC4671616; doi:10.1371/journal.pone.0144129)
Supplement: S3 Table — (PDF) [file pone.0144129.s006.pdf]

S3 Table: Primer sequences and performance characteristics in target specific assay

Primers	Sequences	Amplicon Location	Amplicon Length (bp)	Efficiency	Error (SE)	R^2	Slope
Ol *gapdh*rt-F<br>Ol *gapdh*rt-R	5'- CGGCAAGCTGATAGTCGATG-3'<br>5'- AGAAACACTCCGGTGGACTC-3'	Chr16	117	1.8	0.09	0.98	-3.9
Ol *hprt*rt-F<br>Ol *hprt*rt -R	5'- TCTGGAGAGGGTGTACATCC -3'<br>5'- GATGTAGTCCAACAGGTCGG -3'	Chr10	154	1.9	0.04	0.99	-3.9
Ol *erk2*rt-F<br>Ol *erk2*rt-R	5'- GCAGCGACAGCAGATAGTTC -3<br>5'- GCCGAGATGTTGTCCAACAG -3"	Scaffold 5487	148	1.9	0.03	0.97	-3.4
Ol *prox1*rt-F<br>Ol *prox1*rt-R	5'- ATCTCACCTTACTCAGGCAG-3’<br>5’-TAAGCATGTTGGAGCTTGGG-3'	Chr24	109	1.8	0.01	0.98	-3.8
Ol pri-miR-181a/chr4rt-F<br>Ol pri-miR-181a/chr4rt-R	5'-GAGTGAATATGAGGGGTTAG -3'<br>5'-CAAGAACAGAGCTGATGGTG-3'	Chr4	108	2	0.03	0.98	-3.27
Ol pri-miR-181b/chr4rt-F<br>Ol pri-miR-181b/chr4rt-R	5'-GCTGCAGTGTGTTGATGGAG-3'<br>5'- GTGAGTTTATACCAACCATG-3'	Chr4	100	2.05	0.04	0.95	-3.2
Ol pri-miR-181a/chr17rt-F<br>Ol pri-miR-181a/chr17rt-R	5'-CATGCTTGTGTTGCACAACC-3'<br>5'-GCTGCGACCCTGAGCTGAGC-3'	Chr17	125	2.1	0.09	0.95	-3
Ol pri-miR-181b/chr17rt-F<br>Ol pri-miR-181b/chr17rt-R	5'-CTGCCTGACTTGTGTCTCAG-3'<br>5'-GAGAGGGGAAAATAGAAGTG-3'	Chr17	100	2.02	0.07	0.98	-3.27
Ol pri-miR-181a/chr9rt-F<br>Ol pri-miR-181a/chr9rt-R	5'- CAATGAATGTCCGAATGTCTC-3'<br>5'- GCGGAATCCAGTTTTTGTAG-3'	Chr9	97	2.03	0.07	0.97	-3.2
Ol pri-miR-181b/chr9rt-F<br>Ol pri-miR-181b/chr9rt-R	5'- GCAAGCTGCTCTATGAAATG-3'<br>5'- CCTGATAGGGGTTGACCAAC-3'	Chr9	133	2	0.06	0.99	-3.17
Ol pri-miR-181a/U105rt-F<br>Ol pri-miR-181a/U105rt-R	5'- CAAATGAGGCATCAACCACC-3'<br>5'- GACTAGAGGATGGGGCGGTC-3'	Ultracontig 105	102	2	0.03	0.99	-3.3
Ol pri-miR-181b/U105rt-F<br>Ol pri-miR-181b/U105rt-R	5'- TGTGAATTTACTGACCTTTG-3'<br>5'- ACTCGCCAAGAAATATTAAG-3’	Ultracontig 105	96	2.39	0.23	0.9	-2.6
