# Supplementary material for: TGF-β Controls miR-181/ERK Regulatory Network during Retinal Axon Specification and Growth
Source: PLoS One. 2015 Dec 7;10(12):e0144129. doi: 10.1371/journal.pone.0144129 (PMC4671616; doi:10.1371/journal.pone.0144129)
Supplement: S4 Table — (PDF) [file pone.0144129.s007.pdf]

S4 Table: Mean Ct values +/- SEM for all genes analyzed in each qRT-PCR experiment.

**Graph Fig.2d**		**Experiment1 Ct ± SEM**	**Experiment2 Ct ± SEM**	**Experiment3 Ct ± SEM**
**Control Sample**	*gapdh*	21.01 ± 0.02	22.31 ± 0.06	20.44 ± 0.05
	hprt	23.18 ± 0.03	24.21 ± 0.05	22.54 ± 0.06
	Pri-miR-181a Chr4	27.48 ± 0.02	28.43 ± 0.10	29.28 ± 0.05
	Pri-miR-181b Chr4	23.51 ± 0.01	24.70 ± 0.02	26.54 ± 0.04
	Pri-miR-181a Chr17	28.02 ± 0.01	28.42 ± 0.04	28.30 ± 0.08
	Pri-miR-181b Chr17	24.00 ± 0.13	26.59 ± 0.12	27.81 ± 0.09
	Pri-miR-181a Chr9	28.77 ± 0.02	30.52 ± 0.05	30.56 ± 0.20
	Pri-miR-181b Chr9	24.88 ± 0.03	27.20 ± 0.09	28.35 ±0.05
	Pri-miR-181a Ultr.105	27.48 ± 0.08	28.16 ± 0.07	29.67 ± 0.06
	Pri-miR-181b Ultr.105	33.02 ± 0.08	33.26 ± 0.08	31.39 ± 0.01
**Control+TGFβ Sample**	*gapdh*	20.90 ± 0.10	21.57 ± 0.04	20.65 ± 0.16
	hprt	22.98 ± 0.07	23.56 ± 0.03	22.75 ± 0.12
	Pri-miR-181a Chr4	27.45 ± 0.07	28.24 ± 0.15	29.95 ± 0.08
	Pri-miR-181b Chr4	23.64 ± 0.04	24.33 ± 0.07	27.15 ± 0.01
	Pri-miR-181a Chr17	27.85 ± 0.03	28.35 ± 0.05	28.83 ± 0.01
	Pri-miR-181b Chr17	23.94 ± 0.09	25.92 ± 0.04	27.99 ± 0.07
	Pri-miR-181a Chr9	28.48 ± 0.02	29.73 ± 0.04	31.23 ± 0.07
	Pri-miR-181b Chr9	24.52 ± 0.03	26.14 ± 0.03	28.33 ± 0.04
	Pri-miR-181a Ultr.105	27.41 ± 0.06	27.87 ± 0.03	29.96 ± 0.05
	Pri-miR-181b Ultr.105	32.68 ± 0.03	32.60 ± 0.21	31.46 ± 0.09
**Graph Fig.2e**				
**Control Sample**	*gapdh*	20.08 ± 0.08	20.27 ± 0.02	20.34 ± 0.04
	hprt	23.18 ± 0.01	22.01 ± 0.04	23.98 ± 0.02
	prox1	27.90 ± 0.03	27.20 ± 0.05	29.75 ± 0.07
	erk2	24.80 ± 0.18	27.70 ± 0.09	27.20 ± 0.01
**Control+SB43152 Sample**	*gapdh*	19.80 ± 0.11	19.98 ± 0.03	20.57 ± 0.03
	hprt	22.83 ± 0.03	21.65 ± 0.04	24.30 ± 0.06
	prox1	26.99 ± 0.05	26.72 ± 0.05	29.77 ± 0.05
	erk2	24.24 ± 0.05	26.56 ± 0.06	26.59 ± 0.04
**Control+TGFβ Sample**	*gapdh*	20.17 ± 0.07	19.65 ± 0.07	20.68 ± 0.06
	hprt	23.19 ± 0.04	21.36 ± 0.01	24.26 ± 0.17
	prox1	29.28 ± 0.11	27.98 ± 0.03	31.18 ± 0.05
	erk2	26.27 ± 0.13	27.94 ± 0.14	28.48 ± 0.19
**Graph Fig.5a**				
**Control Sample**	*gapdh*	20.12 ± 0.14	19.64 ± 0.02	19.99± 0.01
	hprt	22.34 ± 0.07	23.01 ± 0.07	22.41 ± 0.07
	prox1	27.91 ± 0.06	27.56 ± 0.06	25.55 ± 0.08
	erk2	27.60 ± 0.09	25.35 ± 0.01	25.85 ± 0.04
**MO-miR-181a/b Sample**	*gapdh*	20.19 ± 0.01	20.66 ± 0.06	20.65 ± 0.02
	hprt	22.50 ± 0.06	23.97 ± 0.05	23.15 ± 0.01
	prox1	27.67 ± 0.18	27.93 ± 0.09	25.75 ± 0.03
	erk2	26.87 ± 0.09	25.72 ± 0.22	25.35 ± 0.02
**MO-miR-181a/b+TGFβ Sample**	*gapdh*	20.08 ± 0.03	20.02 ± 0.06	20.15 ± 0.07
	hprt	22.41± 0.05	23.34 ± 0.07	22.48 ± 0.08
	prox1	28.15 ± 0.17	28.12 ± 0.13	25.86 ± 0.17
	erk2	27.92 ± 0.16	25.97 ± 0.15	26.46 ± 0.13
**Graph Fig.6a**				

**Control Sample**	*gapdh*	20.05 ± 0.01	20.53 ± 0.01	20.15 ± 0.01
	hprt	23.76 ± 0.01	23.00 ± 0.01	23.89 ± 0.01
	prox1	27.75 ± 0.05	27.22 ± 0.03	27.67 ± 0.04
	erk2	26.68 ± 0.09	25.54 ± 0.11	26.59 ± 0.09
**MO-protector-erk2 Sample**	*gapdh*	20.41 ± 0.04	20.90 ± 0.01	20.51 ± 0.04
	hprt	23.97 ± 0.09	23.19 ± 0.02	24.13 ± 0.09
	prox1	28.04 ± 0.07	27.76 ± 0.02	28.28 ± 0.07
	erk2	26.41 ± 0.12	25.23 ± 0.07	26.47 ± 0.12
**MO- protector-erk2 +TGFβ Sample**	*gapdh*	20.77 ± 0.01	20.75 ± 0.02	20.91 ± 0.01
	hprt	24.39 ± 0.09	23.23 ± 0.21	24.61 ± 0.09
	prox1	29.17 ± 0.02	28.57 ± 0.01	29.63 ± 0.02
	erk2	26.97 ± 0.02	25.23 ± 0.08	26.67 ± 0.02
**Graph Fig.S1a**				
**Control Sample**	*gapdh*	20.05 ± 0.01	20.53 ± 0.01	20.15 ± 0.01
	hprt	23.76 ± 0.01	23.00 ± 0.01	23.89 ± 0.01
	prox1	27.75 ± 0.05	27.22 ± 0.03	27.67 ± 0.04
	erk2	26.68 ± 0.09	25.54 ± 0.11	26.59 ± 0.09
**MO-protector-erk2 Sample**	*gapdh*	20.41 ± 0.04	20.90 ± 0.01	20.51 ± 0.04
	hprt	23.97 ± 0.09	23.19 ± 0.02	24.13 ± 0.09
	prox1	28.04 ± 0.07	27.76 ± 0.02	28.28 ± 0.07
	erk2	26.41 ± 0.12	25.23 ± 0.07	26.47 ± 0.12
