# Supplementary material for: TGF-β Controls miR-181/ERK Regulatory Network during Retinal Axon Specification and Growth
Source: PLoS One. 2015 Dec 7;10(12):e0144129. doi: 10.1371/journal.pone.0144129 (PMC4671616; doi:10.1371/journal.pone.0144129)
Supplement: S5 Table — (PDF) [file pone.0144129.s008.pdf]

S5 Table: **qRT-PCR thermocycling conditions**

	Temperature	Time	Ramp.Rate (°C/s)	Cycles
**Pre-incubation**	95°C	5 min	4.4	1
**Amplification**	95°C<br>60°C<br>72°C	10 sec<br>10 sec<br>15 sec	4.4<br>2.2<br>4.4	45
**Melt curve**	95°C<br>65°C<br>97°C	5 sec<br>1m	4.4<br>2.2<br>0.11	1
**Cooling**	40°C	20 sec	1.5	1
