# Supplementary material for: TGF-β Controls miR-181/ERK Regulatory Network during Retinal Axon Specification and Growth
Source: PLoS One. 2015 Dec 7;10(12):e0144129. doi: 10.1371/journal.pone.0144129 (PMC4671616; doi:10.1371/journal.pone.0144129)
Supplement: S6 Table — (PDF) [file pone.0144129.s009.pdf]

S6 Table: Mature miRNA quantitative assay thermocycling conditions

	Temperature	Time	Ramp.Rate (°C/s)	Cycles
**Pre-incubation**	95°C	10 min	4.4	1
**Amplification**	95°C<br>60°C	15 sec<br>1 min	4.4<br>2.2	45
**Cooling**	40°C	30 sec	2	1
