# Supplementary material for: TGF-β Controls miR-181/ERK Regulatory Network during Retinal Axon Specification and Growth
Source: PLoS One. 2015 Dec 7;10(12):e0144129. doi: 10.1371/journal.pone.0144129 (PMC4671616; doi:10.1371/journal.pone.0144129)
Supplement: S7 Table — (PDF) [file pone.0144129.s010.pdf]

S7 Table: Western blotting conditions used for each antibody

Antibody	Dilution	Supplier
Total ERK1/2	1:1000	Cell Signaling
Phospho-ERK1/2	1:1000	Cell Signaling
RhoA	1:200	Millipore
GAPDH	1:500	Santa Cruz
